# Supplementary material for: Constrained CMIP6 projections indicate less warming and a slower increase in water availability across Asia
Source: Nat Commun. 2022 Jul 15;13:4124. doi: 10.1038/s41467-022-31782-7 (PMC9287300; doi:10.1038/s41467-022-31782-7)
Supplement: Supplementary file 1 — Supplementary Information [file 41467_2022_31782_MOESM1_ESM.pdf]

Supplementary Information for

Constrained CMIP6 projections indicate less warming and a slower increase in water availability across Asia

Authors: Yuanfang Chai[1,2], Yao Yue[1,3*], Louise J. Slater[4], Jiabo Yin[1], Alistair G.L. Borthwick[5,6], Tiexi Chen[7], Guojie Wang[7].

Affiliations:

1 State Key Laboratory of Water Resources and Hydropower Engineering Science, Wuhan University, Wuhan, 430072 China

2 Vrije Universiteit Amsterdam, Department of Earth Sciences, Boelelaan 1085, 1081 HV Amsterdam, the Netherlands

3 Institute for Water-Carbon Cycles & Carbon Neutrality, Wuhan University, Wuhan 430072 China

4 Institute for Infrastructure and Environment, School of Geography and the Environment, University of Oxford, Oxford, OX1 3QY, United Kingdom

5 School of Engineering, The University of Edinburgh, The King's Buildings, Edinburgh EH9 3JL, UK

6 School of Engineering, Mathematics and Computing, University of Plymouth, Drake Circus, Plymouth PL4 8AA, UK

7 School of Geographical Sciences, Nanjing University of Information Science and Technology, Nanjing 210044, China.

***Corresponding author**: yueyao@whu.edu.cn

Supplementary Text

1. Comparison between CMIP5 and CMIP6 models in reproducing historical temperature and precipitation

Land surface air temperature across Asia during 1970–2014 is very well simulated by CMIP6 models relative to observations, with an error of -0.01 °C (4.43 °C calculated from average grid cell values of all raw CMIP6 models, and no bias correction performed as opposed to 4.44 °C for the observational HadCRUT4 data set). By comparison, the discrepancy between CMIP5 outputs and the observations is much higher (-0.34 °C). Here we found that the CMIP6 models perform better (mean absolute error of 1.52 °C) than the CMIP5 models (mean absolute error of 1.77 °C). Spatial distributions of the CMIP6-based difference between simulated historical temperature and observed temperature (Supplementary Fig. 2a) and the CMIP5-based difference (Supplementary Fig. 2b) during the mutual historical period of CMIP5 and CMIP6 (i.e. 1986–2005) also demonstrate that the outputs of the CMIP6 models are generally better than those of the CMIP5 models, especially for the Himalaya Mountains and North India.

In reproducing historical precipitation, the performance of CMIP6 models is also better than CMIP5 models when comparing the mean absolute bias (0.432 mm day^{-1} vs. 0.441 mm day^{-1}), even though the mean bias of the CMIP5 models is smaller than that of the CMIP6 models (0.23 mm day^{-1} vs. 0.32 mm day^{-1}). Spatial distributions of the CMIP6-based difference between simulated historical temperature and observed temperature (Supplementary Fig. 2c) and the CMIP5-based difference (Supplementary Fig. 2d) during the mutual historical period of CMIP5 and CMIP6 (i.e. 1986–2005) also demonstrate that the CMIP6 models generally perform better than the CMIP5 models. This is especially the case for the central and northern areas of India, the eastern area of Southeast Asia, and Southeast China.

Overall, the CMIP6 models perform better at reproducing historical temperature and precipitation in Asia because the latest generation of ESMs (CMIP6) has finer vertical and horizontal spatial resolutions, and includes more comprehensive designs of numerical experiments and more detailed process descriptions.

2. Influence of dynamic factors on the relationship between precipitation and temperature in Asia

Previous studies mainly classified factors affecting precipitation into ‘dynamic’ and ‘thermodynamic’ categories[1-3]. By considering the dependency of precipitation change on regional atmospheric circulation, we found that dynamic factors may not significantly affect long-term average changes in precipitation over large regions such as Asia.

Two variables have been widely used to measure the dynamic conditions, i.e., pressure vertical velocity as an indicator of the strength of dynamic disturbance[1-2] and convective available potential energy (CAPE) as an indicator of atmospheric instability and convection strength[4]. These two variables are commonly used to explore atmospheric dynamic impacts on precipitation extremes. We therefore attempt to explore whether these drivers govern long-term changes in mean precipitation. First, we test the relationship between annual precipitation and annual mean pressure vertical pressure velocity at 11 pressure levels in Asia based on the ERA5 reanalysis data during 1979-2014 (https://cds.climate.copernicus.eu/cdsapp#!/dataset/reanalysis-era5-pressure-levels-monthly-means?tab=overview), and find some correlation (not strong, see Supplementary Fig. 8, $-0.46<r<-0.26$, $p>0.01$). Then, we also test the CAPE relationship with annual precipitation in Asia during 1979-2014 (https://cds.climate.copernicus.eu/cdsapp#!/dataset/reanalysis-era5-single-levels-monthly-means?tab=overview), but find poor correlation (Supplementary Fig. 9, r=-0.24, $p>0.1$). These results imply that the ‘dynamic’ factors do not strongly affect long-term mean precipitation over the large area considered in this study as the ‘thermodynamic’ factors. However, it should be noted that the correlations between vertical pressure velocity and precipitation may be significant at the 95% level at certain pressure levels when $|r|$ is around 0.5. Therefore, it would be worthwhile to determine the specific contributions of the dynamic factors to the long-term trend in precipitation at the continental scale in future work.

Emori and Brown (2005)[2] suggested that dynamic change provides only a partial explanation for the increase in annual mean precipitation in the tropical Pacific region (belonging to its oceanic area), whereas thermodynamic change explains almost all the increase at mid- to high-latitudes (including Asia) based on model experiments for 2081-2100. A stochastic simulation of large-scale condensation[5], based on a partial differential equation that includes water vapor transport and thermodynamics processes (Equation 11 in O’Gorman and Schneider, 2008[5]), showed that

large-scale (i.e. the grid scale used by O'Gorman and Schneider, 2008[5]) precipitation increases approximately linearly with temperature over a wide range of temperatures (Figure 7 in O'Gorman and Schneider, 2008[5]). We acknowledge that the 'dynamic' factors play an important role in altering precipitation extremes at regional scale[1]. Nonetheless, our study focuses on long-term changes in mean precipitation; previous studies have highlighted divergent responses of mean and extreme precipitation to climate change[2]. At the time of writing, it is difficult to find evidence to support the proposition that dynamic factors significantly drive such long-term changes in mean precipitation over continental Asia. Given that the 'thermodynamic' factors have been widely recognized as playing the lead role in driving changes in long-term mean precipitation over large areas[2,5], we leave exploration of the effect of 'dynamic' attributions to future work.

Large-scale patterns of climate variability including monsoons and teleconnections (such as the El Niño Southern Oscillation, and Arctic Oscillation) which are closely related to atmospheric circulation also influence the climate in Asia[6-7]. Thus, we also analyze whether the relationship between the growth rates of simulated historical temperature and projected future precipitation in Asia is significantly affected by regions experiencing strong atmospheric circulation. According to Van der Ent et al. (2010)[8], precipitation in regions with low continental precipitation-recycling ratio (ρ_c, defined as the ratio of precipitation of continental origin [not necessarily from the same continent] to that of both continental and oceanic origin) primarily derives from moisture transport from the oceans. Therefore, we selected areas with $\rho_c < 0.5$ in Asia as regions subject to monsoons and ENSO events (such as southeastern China, etc.). By examining the relationship between the growth rates of simulated historical temperature and projected future precipitation when excluding regions of low ρ_c, we found that the linear regression results remain almost unchanged for all four shared socioeconomic pathways (SSPs) (i.e., changes in the regression slope are only 0.2–16.0%, see Supplementary Fig. 10). This implies that the influence of regions with strong atmospheric circulation is very small in Asia.

We examined the historical precipitation-temperature relations using a moving average method with window lengths of 5–10 years to reduce the influence of interannual variability[9,10]. The results show that strong positive relations still exist between precipitation and surface air temperature after smoothing out extreme fluctuations (Supplementary Fig. 11a), supporting the reliability of the

previously identified relationship. Furthermore, the sensitivity of precipitation to temperature change estimated by the new relationship remains (only slightly increased by 8.9–10.1%, see Supplementary Fig. 11b), suggesting that the effects of monsoons and climate variability on the long-term relationship between precipitation and temperature are not significant.

3. Influence of regions with negative correlation coefficients on the emergent constraint relationships

Certain regions with a negative relationship between temperature and precipitation (e.g., Pakistan and Indonesia, see Supplementary Fig. 7) are included when setting up the emergent constraint relationship to reflect better the complexity of the atmosphere-land-ocean interactions across Asia. Given that these regions only occupy 7.9–18% (depending on SSP) of the total area of Asia, we find that such a small percentage area does not affect the area-averaged emergent constraint relationship. We analysed the effect of including regions with a negative relationship between temperature and precipitation by examining the emergent constraint relationship both with and without these regions. We found that positive emergent constraint relationships remained for all four SSPs, with changes in regression slopes of only 2.8–4.0% (Supplementary Fig. 12), indicating that the influence of such regions on the emergent constraint relationship is relatively small.

4. Reasonability of constraining future temperature growth rate based on constrained results of future precipitation growth rate

Considering the non-negligible bias (up to 11.35%) between the simulated (0.363 ± 0.0732 °C decade^{-1}) and the observed (0.326 ± 0.035 °C decade^{-1}) annual growth rate in temperature (Supplementary Fig. 25), it is necessary to constrain the projected future temperature growth rate. The rationale behind the reducing uncertainty in future temperature growth rate based on constrained precipitation results is as follows.

First, we find a good correlation between annual precipitation growth rate and sensitivity of temperature to latent heat flux based on CMIP6 model simulations during 1970-2014 (Supplementary Fig. 26, r=0.61, p<0.01), indicating that wet (dry) models tend to have higher (lower) temperature sensitivity to latent heat flux. This demonstrates that the CMIP6 models can

satisfactorily capture precipitation feedback, because more water vapor in wetter condition imposes a stronger warming effect on temperature. We acknowledge that several other factors may also affect precipitation, but have been omitted from our study due to lack of data. For example, precipitation events are associated with thick cloud that cools the surface by increasing shortwave reflectance[11]. We recommend future study be carried out on this feedback effect on temperature.

In addition to the above mechanism of precipitation feedback to the temperature trend, we also note from previous studies that once an emergent constraint between factors A and B has been confirmed through plausible mechanisms, the emergent constraint can be used either to constrain the uncertainty in A based on B measurements, or to constrain the uncertainty in B based on A measurements. For example, Wenzel et al. (2016)[12] found that change in gross primary production (GPP) is the main reason for the change in amplitude of the CO_2 seasonal cycle. Wenzel et al. then used the observed amplitude of the CO_2 seasonal cycle (i.e. the result of GPP change) to constrain the annual mean GPP (i.e. the driver behind CO_2 variation). This holds because once a relationship $\boldsymbol{f}$ linking A and B (i.e. B = $\boldsymbol{f}$(A), in Wenzel et al.'s case, amplitude of CO_2 = $\boldsymbol{f}$(GPP change)[12]) has been identified, we can either generate B using A through $\boldsymbol{f}$, or else infer A from B through the inverse relationship $\boldsymbol{f}^{-1}$ (GPP change = $\boldsymbol{f}^{-1}$(amplitude of CO_2)). Our approach is similar to that of Wenzel et al. (2016)[12]. Noting that temperature change is the dominant factor driving precipitation spread, we can then constrain the future temperature growth rate using the constrained results of precipitation which exhibit less spread than the raw projections. This holds because when spread in future precipitation growth rate is reduced, a corresponding spread reduction in future temperature growth rate must also occur due to their linkage identified in the CMIP6 ensembles.

Taking the above into account, we therefore believe reducing the future temperature growth rate by the constrained precipitation growth rate is reasonable.

5. Direction and range of variations in the constrained results

In Figure 3b, d, f, we find that inter-model uncertainty decreased effectively after applying the emergent constraint. The upper and lower lines of the colour histograms stand for mean ± one standard deviation, respectively, with constrained standard deviation (i.e. 0 < SD' < SD). When the mean value shifts downwards (i.e. mean' < mean, as in Figure 3b and d), the lower line (indicating

mean – one standard deviation) descends less than the upper line (representing mean + one standard deviation), because |(mean'-SD')-(mean-SD)| < |(mean'+SD')-(mean+SD)|. Similarly, when the mean value shifts upwards (i.e. mean' > mean, as in Figure 3f), the upper line ascends less than the lower.

Model selection might also affect the variation range of different climate variables. Therefore, we examined the effect of model selection by selecting the same models involved in developing relationships between future total evaporation/snow cover fraction growth rate and future precipitation growth rate (listed in Supplementary Table 3), and then derived new constraint relationships (see Supplementary Fig. 28). The similarity between the results shown in Supplementary Fig. 28b,d and Figure 3d,f implies that model selection does not change the relative variation range of (mean + one standard deviation) to that of (mean – one standard deviation).

6. Comparison between emergent constraint and bias correction methods

Both emergent constraint and bias correction methods essentially consider the difference between projections and observations; they both assume that the difference between projections and observations over a historical period is likely to be the same in the future. However, we believe the major advantage of the emergent constraint method is that it is more physically plausible by assuming that the physics (i.e., the relationship between different variables) remains the same in the historical and future periods, whereas most bias correction methods simply apply a "shift" to the data. In implementing the emergent constraint method, we cannot claim that we identify an emergent constraint relationship without the support of physical mechanisms, even though we find a tight relationship between the simulated historical changes of one climate variable and the projected future changes of another climate variable[13] . By contrast, bias correction methods have been developed to adjust or downscale simulated climate variables[14-15] based on correction factors obtained by simply exploring the statistical differences between simulations and observations.

Without sufficient emphasis on the physical mechanisms, bias correction methods sometimes destroy the physical consistency among different climate variables, leading to failure in correcting the simulated results. For instance, temperature may become sub-zero after bias correction, but the

rainfall is not automatically converted into snowfall[16]. Thus, the emergent constraint method is likely to be more reliable than bias correction methods.

Supplementary Figures

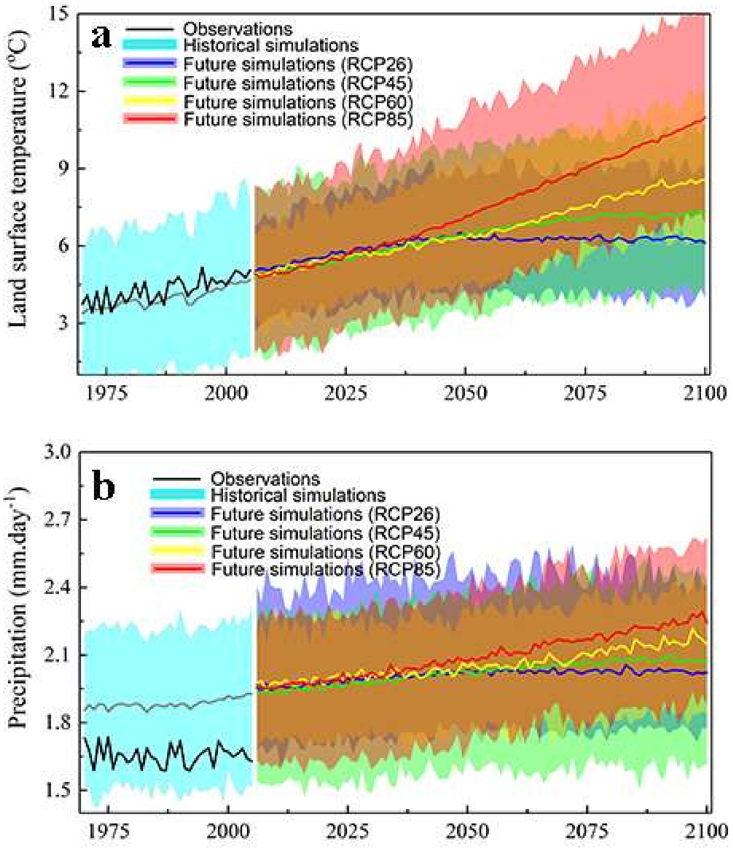

Supplementary Fig. 1. Trends in land surface temperature and precipitation in Asia based on the CMIP5 simulations and observations. (**a**) and (**b**) are the changes in land surface temperature (°C) and precipitation (mm day^{-1}) for 1970–2100, based on outputs from 46 CMIP5 models. Observations/simulations are shown for 1970–2014, and projections are for 2015–2100.

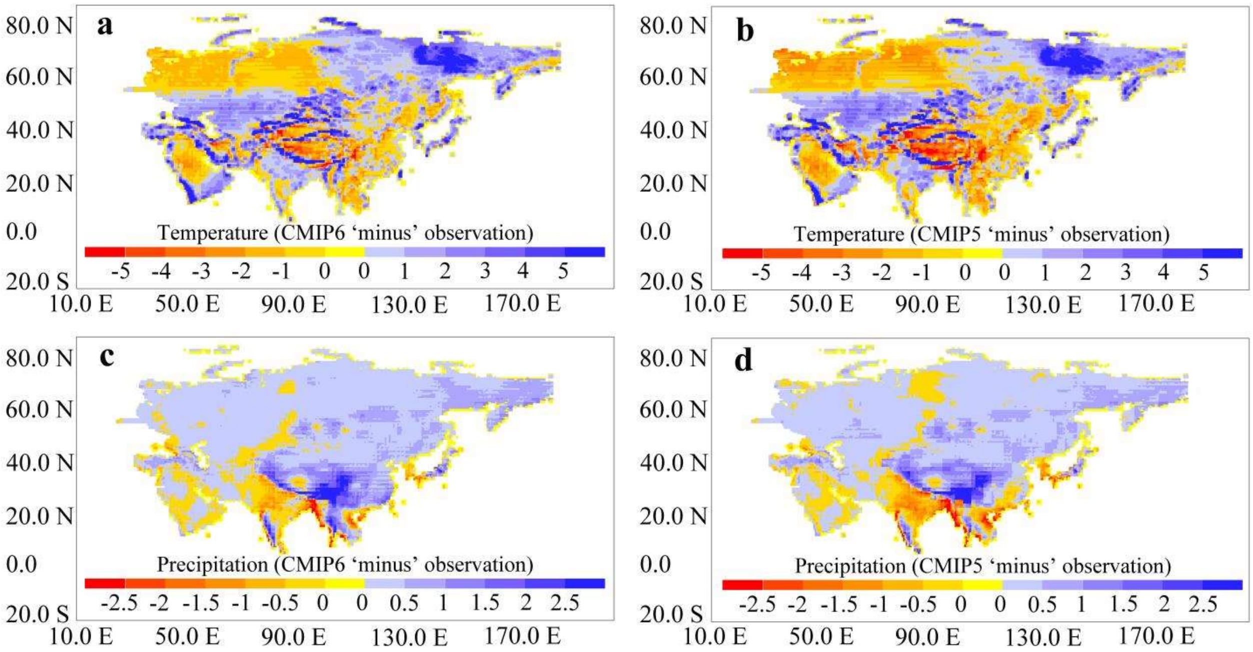

Supplementary Fig. 2. Performance of CMIP6 and CMIP5 models in simulating land surface temperature (℃) and precipitation (mm day^{-1}) compared with HadCRUT4 observations. (**a**) is the CMIP6-based difference between the simulated historical temperature and the observed temperature for 1986–2005. (**b**) is the CMIP5-based difference between the simulated historical temperature and the observed temperature for 1986–2005. (**c**) and (**d**) are the differences in precipitation between CMIP6 and observations, and between CMIP5 and observations, respectively.

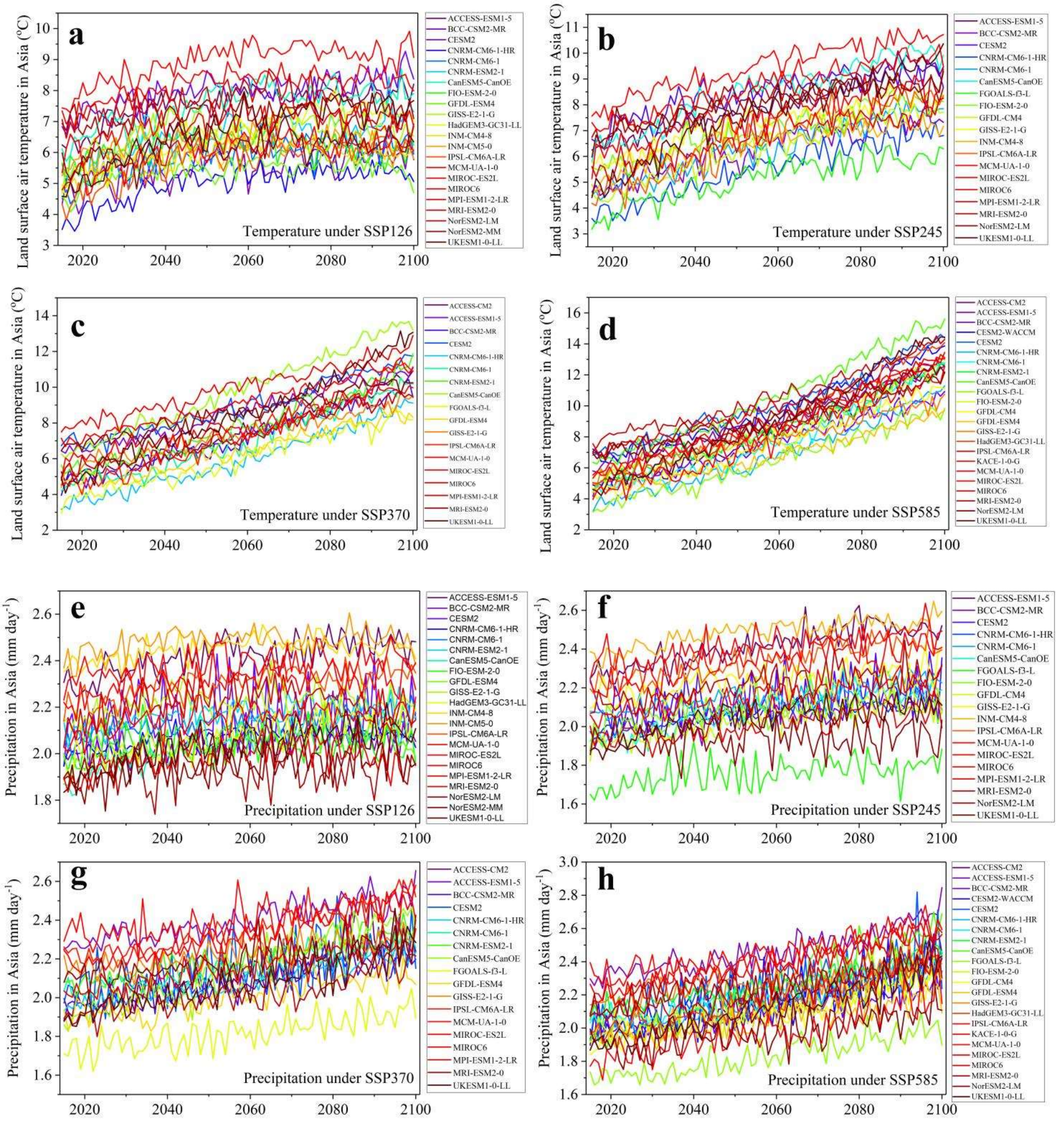

Supplementary Fig. 3. Future trends in temperature and precipitation for Asia during 2015–2100 obtained for each CMIP6 model. (**a**), (**c**), (**e**) and (**g**) are trends in temperature under emission scenarios SSP126, SSP245, SSP370, and SSP585. (**b**), (**d**), (**f**) and (**h**) are trends in precipitation under emission scenarios SSP126, SSP245, SSP370, and SSP585.

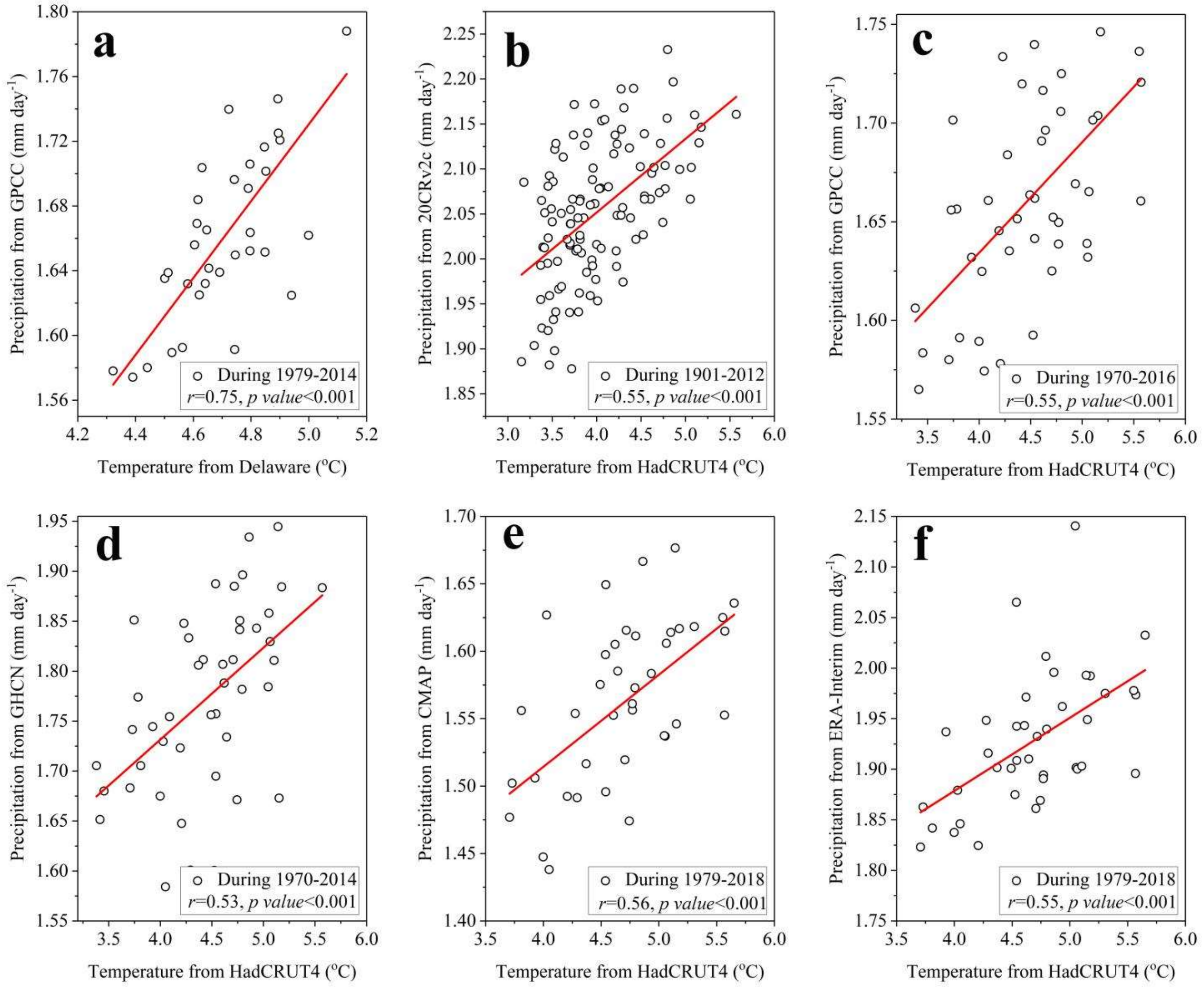

Supplementary Fig. 4. Observed linear relations between temperature and precipitation in Asia based on a series of global precipitation and temperature data sets. Each circle represents one year. Note: temperature from the commonly-used HadCRUT4 data set is used to build the precipitation-temperature relationships in conjunction with precipitation from several other data sets.

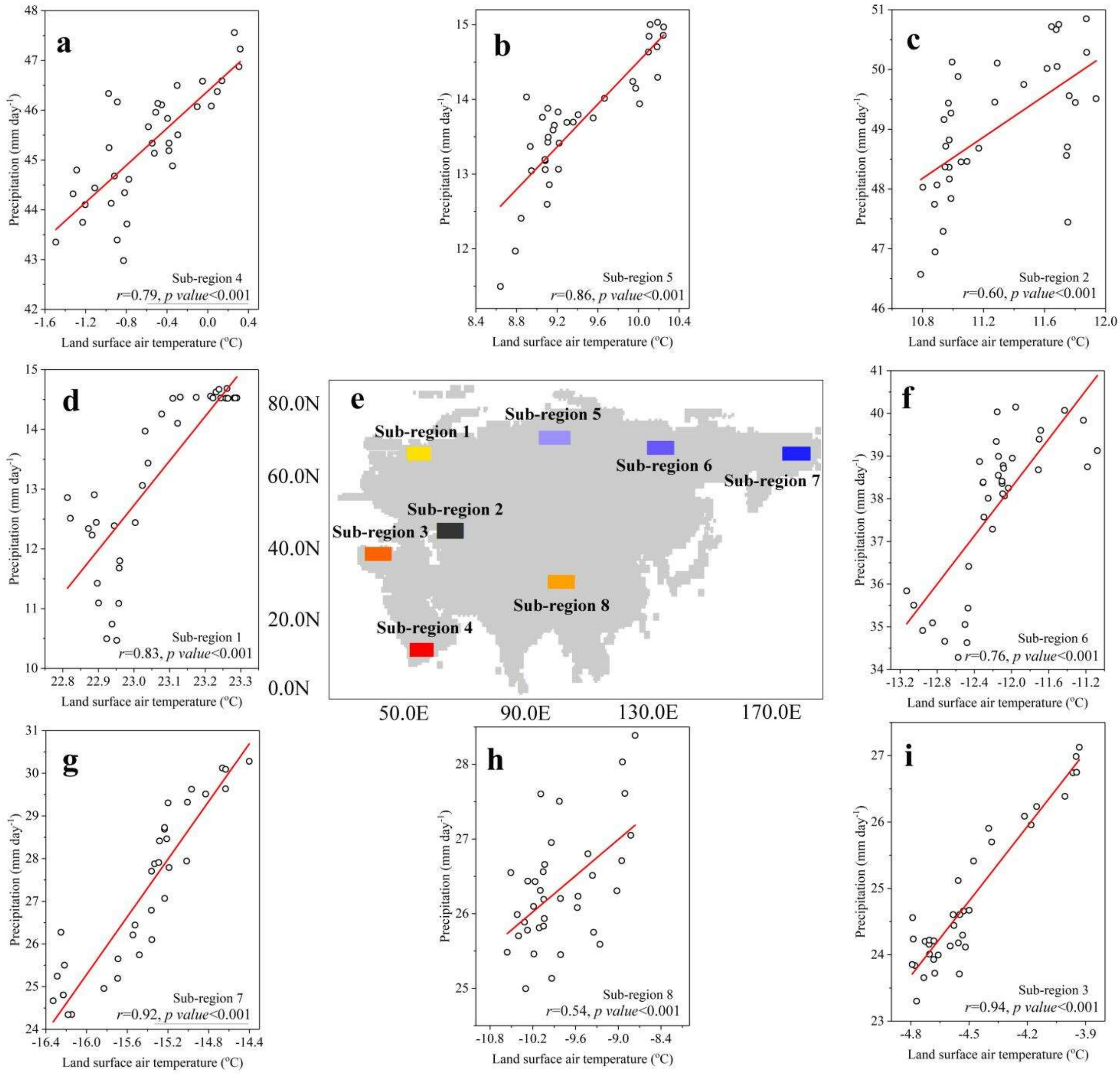

Supplementary Fig. 5. Observed linear relations between temperature and precipitation in eight sub-regions. Based on time series data of temperature and precipitation, both with 10-year moving windows, (**a**), (**b**), (**c**), (**d**), (**f**), (**g**), (**h**) and (**i**) are the positive relationships for sub-regions 1 to 8. Extreme weather conditions driven by monsoons or large-scale climate variability, such as the El Niño Southern Oscillation, may lead to anomalously high precipitation and low temperature. These anomalies may non-linearly affect underlying trends in the Asian climate. After smoothing out extreme fluctuations using moving averages, we obtain a more reliable, accurate relationship between precipitation and surface air temperature in the observations for the entire period (1970–2014). (**e**) shows the locations of the eight randomly-selected sub-regions.

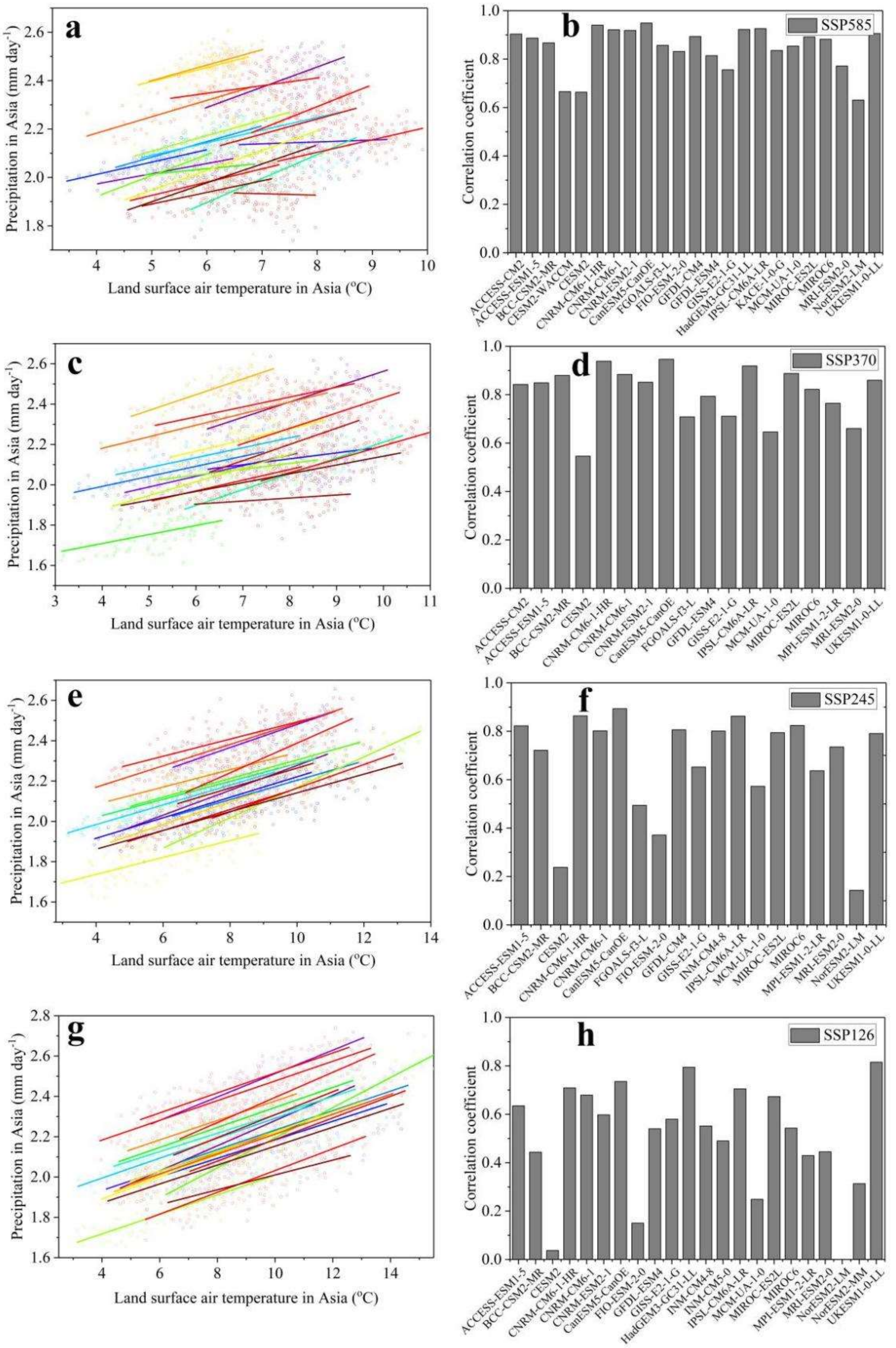

Supplementary Fig. 6. Linear relationships between temperature and precipitation for each CMIP6 model and the associated Pearson's correlation coefficient (R). (**a**), (**c**), (**e**) and (**g**) are linear relationships for the SSP126, SSP245, SSP370, and SSP585 emission scenarios. (**b**), (**d**), (**f**) and (**h**) are correlation coefficients for the SSP126, SSP245, SSP370, and SSP585 emission scenarios.

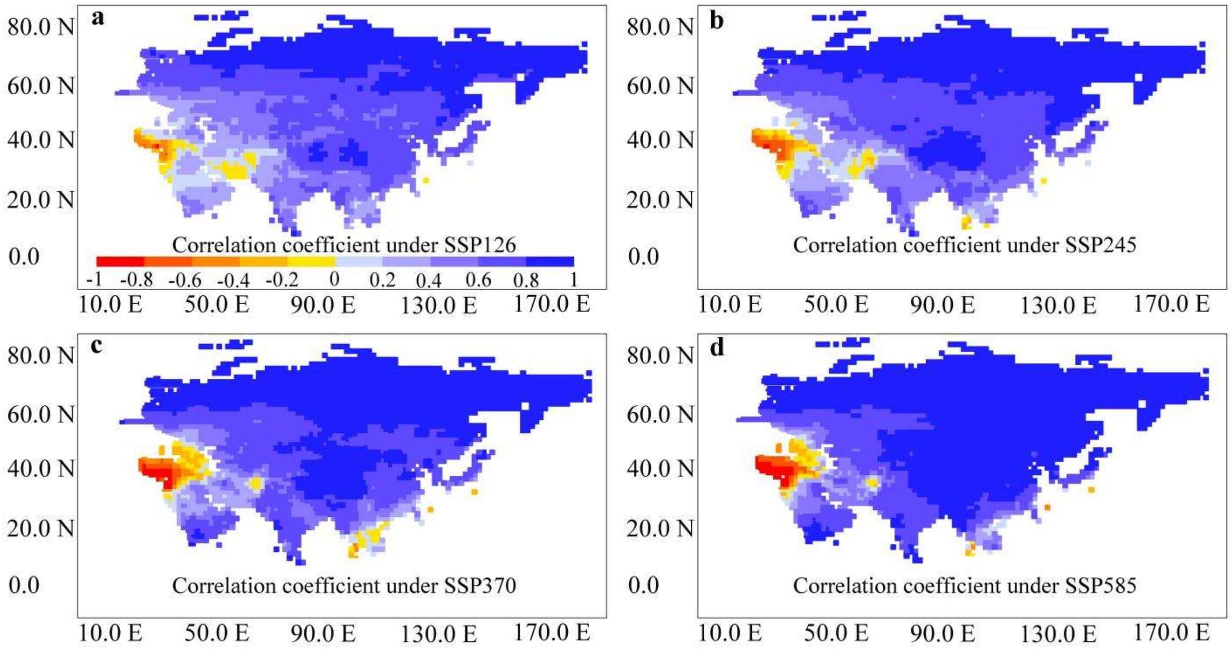

Supplementary Fig. 7. Distribution of correlation coefficients between precipitation and temperature in Asia during 1982–2100 based on CMIP6 ensemble (27 models). (**a**), (**b**), (**c**) and (**d**) are correlation coefficient distributions for SSP126, SSP245, SSP370, and SSP585 emission scenarios. The negative relationship between precipitation and temperature in Pakistan and Indonesia covers only 7.9–18% of Asia, depending on the SSP. Furthermore, complex atmosphere-land-ocean interactions in various sub-regions may lead to a more complicated sensitivity of precipitation to temperature. Therefore, it may be usual (and acceptable) for negative relationships between temperature and precipitation to occur in small sub-regions of Asia. We therefore included these sub-regions in the analysis.

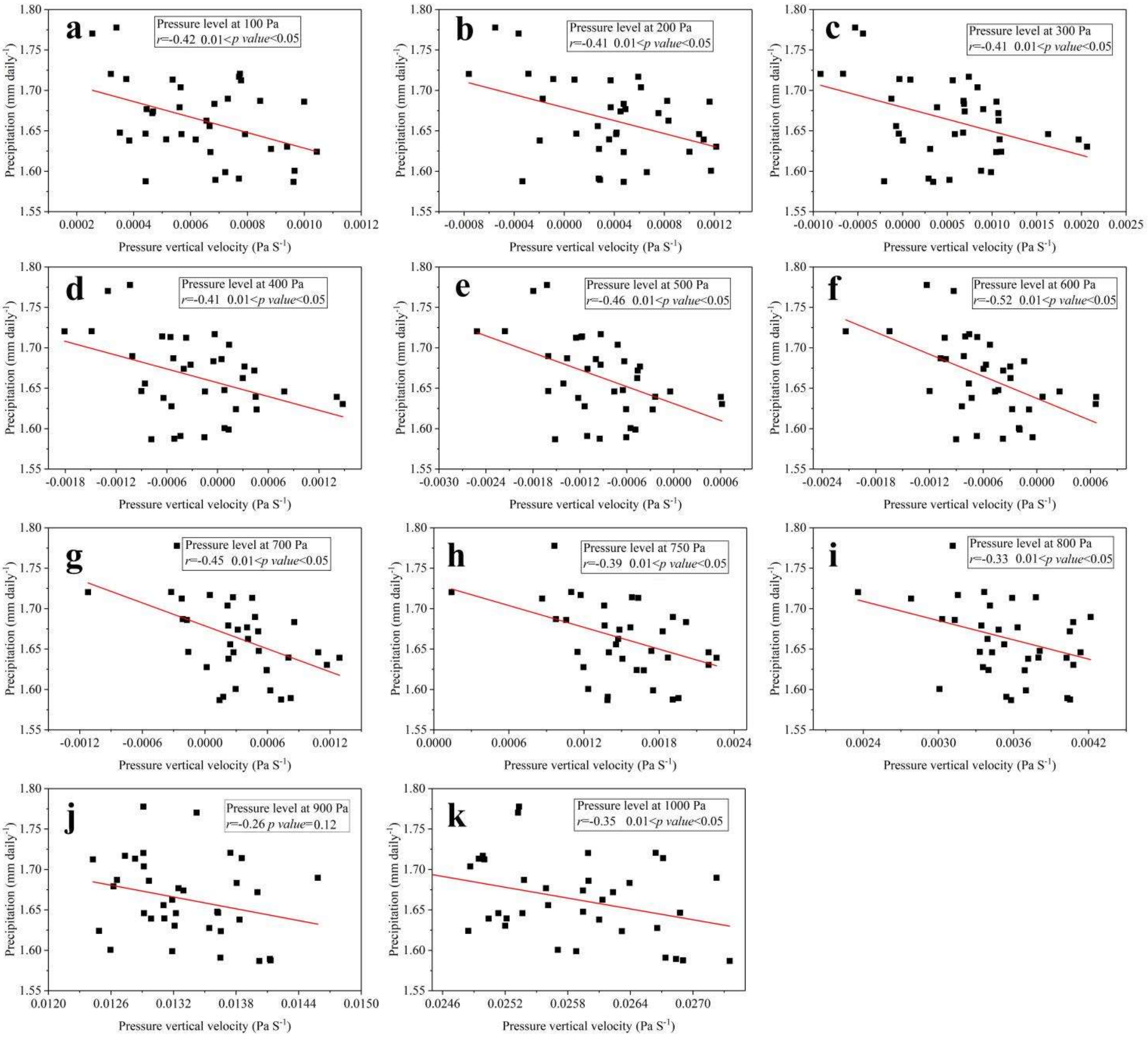

Supplementary Fig. 8. Poor correlation between pressure vertical velocity and precipitation at 11 pressure levels and precipitation in Asia during 1979-2014.

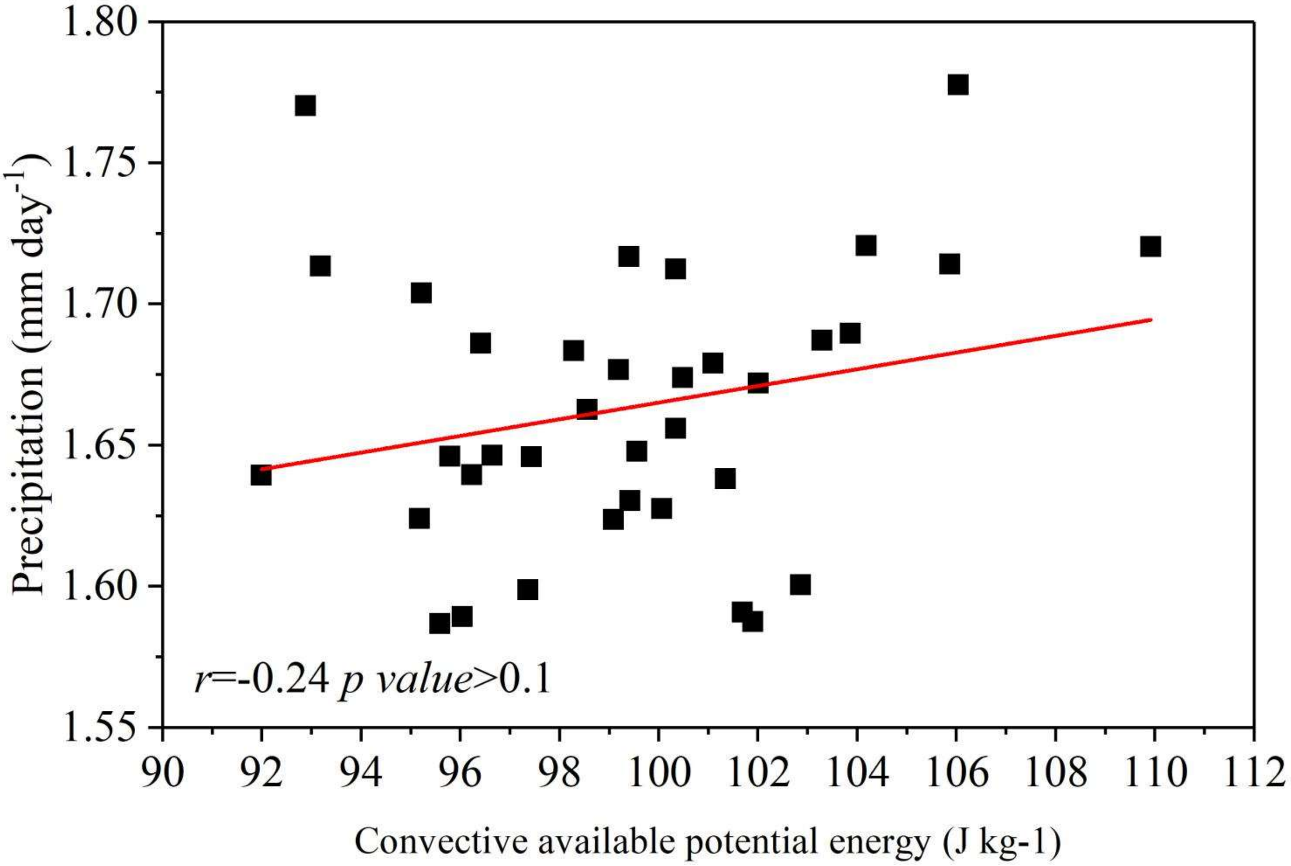

Supplementary Fig. 9. Poor correlation between convective available potential energy and precipitation in Asia during 1979-2014 based on ERA5 dataset.

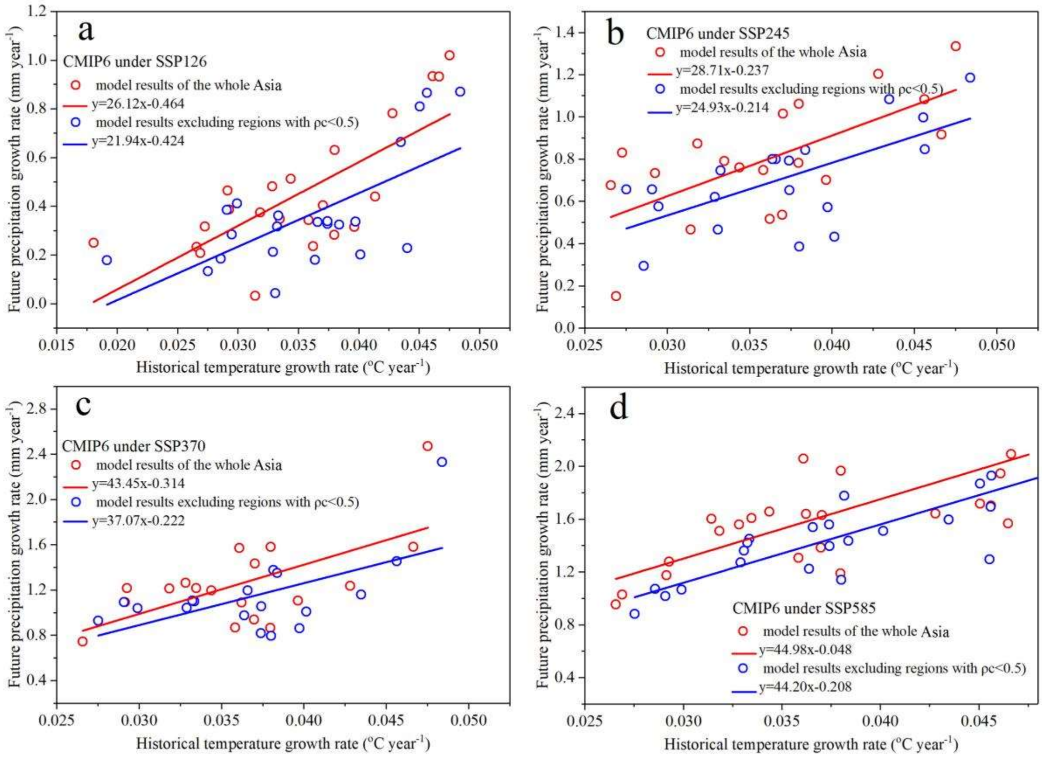

Supplementary Fig. 10. Relationships between historical temperature growth rate and future precipitation growth rate in Asia, with (red fitted lines) and without (blue fitted lines) regions of low precipitation-recycling ratio ($\rho_c <$ 0.5), for the following emission scenarios: (a) SSP126; (b) SSP245; (c) SSP370; and (d) SSP585.

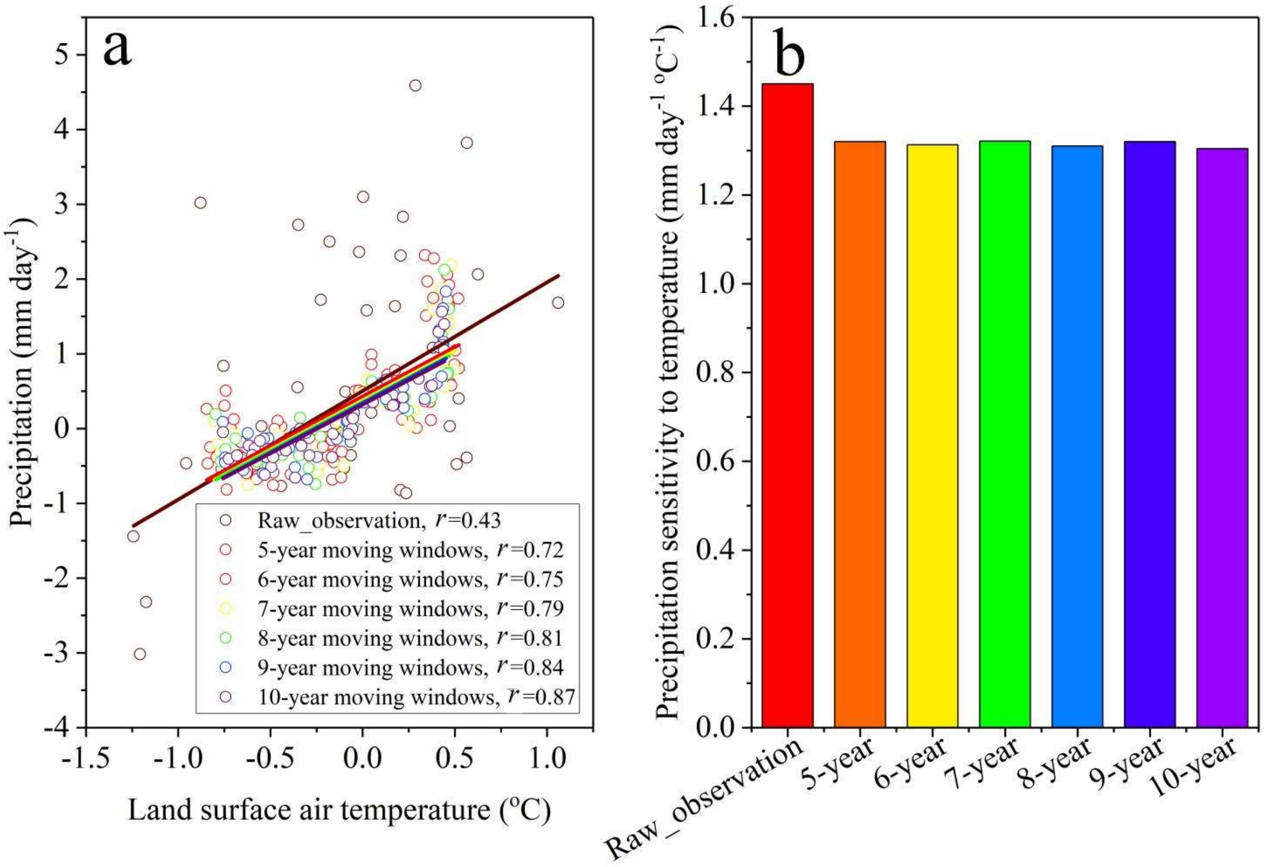

Supplementary Fig. 11. Linear relationships between precipitation and land surface air temperature during 1970–2014. (**a**) presents linear relationships for the initial observations (Raw_observation) and for observations smoothed by a series of moving windows (with window lengths ranging from 5 to 10 years). Each circle represents a year. (**b**) indicates the sensitivity of precipitation to temperature (slope values in **a**) for the initial observations and for observations smoothed by the series of moving windows.

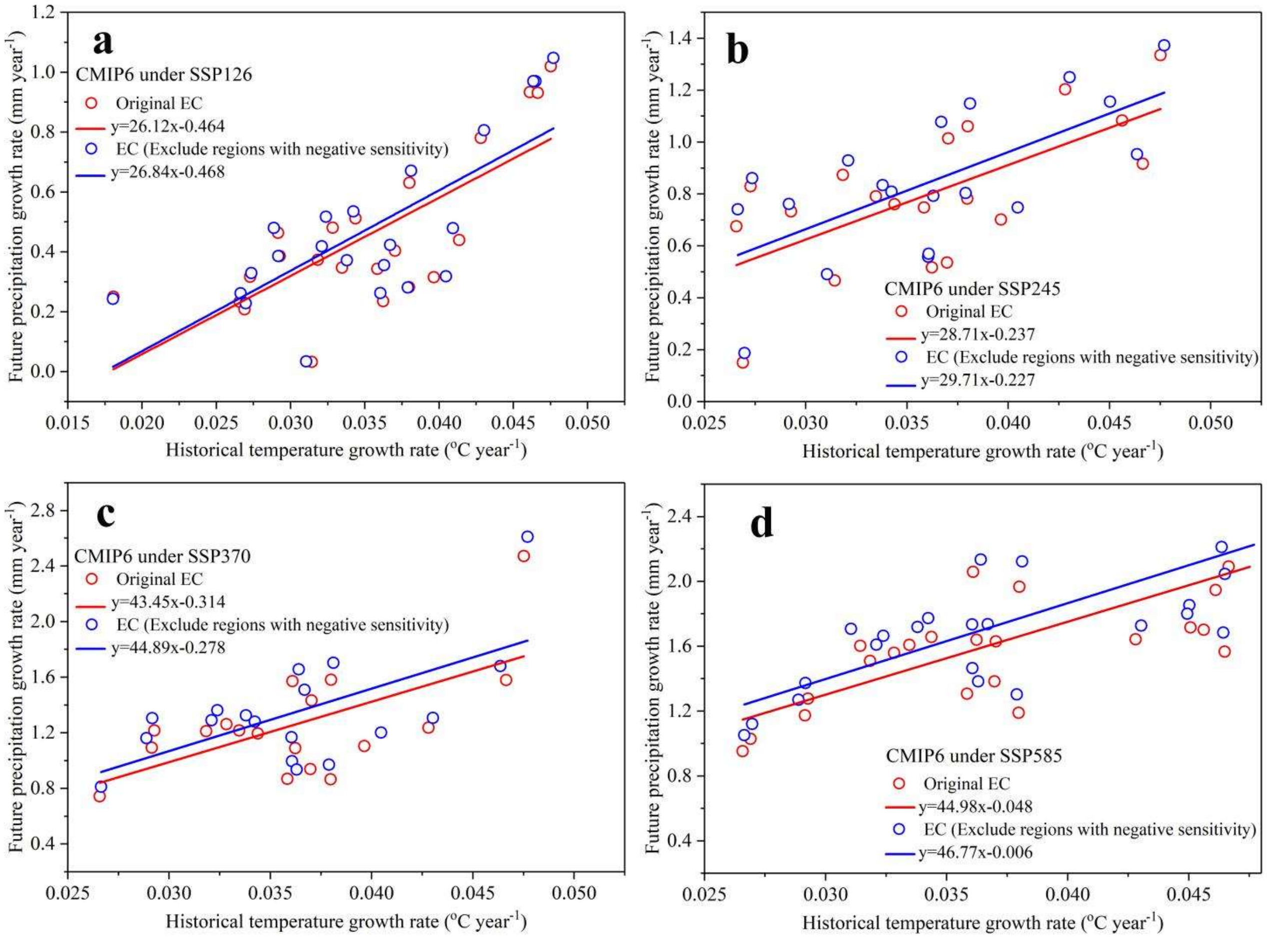

Supplementary Fig. 12. Emergent constraint relationships between historical temperature growth rate and future precipitation growth rate. (**a**), (**b**), (**c**) and (**d**) indicate the emergent constraint relationships for the SSP126, SSP245, SSP370, and SSP585 emission scenarios. Red fitted lines are the constraint relationships for the whole of Asia, including regions with negative relationships between precipitation and temperature. Blue fitted lines are constraint relationships computed by excluding regions with negative relationships.

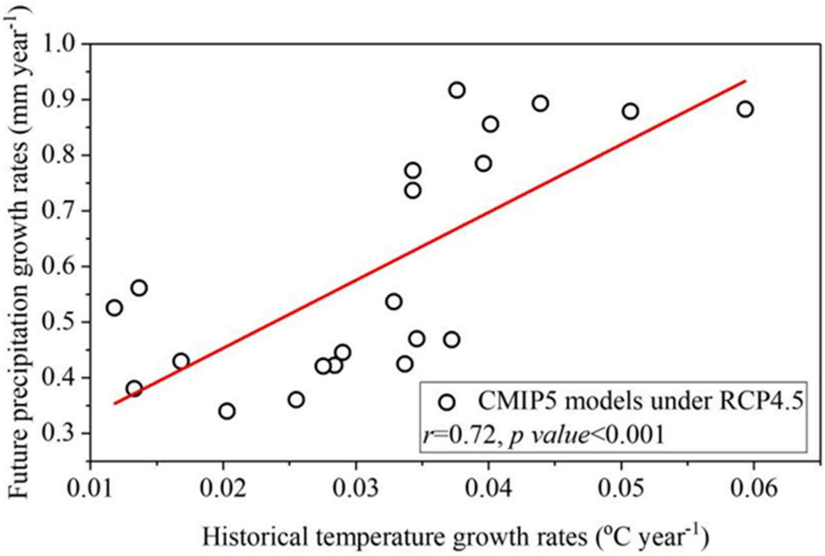

Supplementary Fig. 13. Relationship between historical temperature growth rate and future precipitation growth rate in Asia based on CMIP5 projections under the RCP4.5 emission scenario. Each circle represents a CMIP5 model.

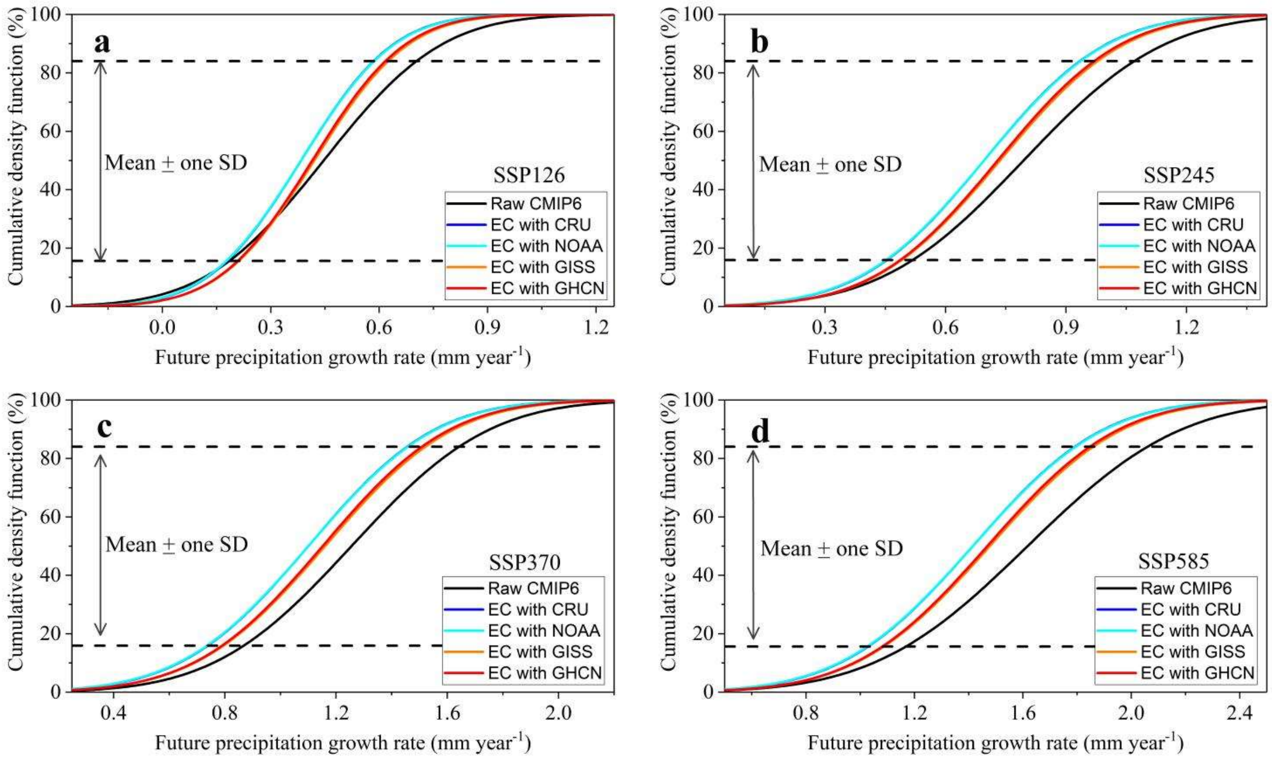

Supplementary Fig. 14. Cumulative density functions for future precipitation growth rates before and after application of the emergent constraint. The black line is the cumulative density function before the emergent constraint. Colored lines are the cumulative density functions after the emergent constraint with a series of observations (CRU, NOAA, GISS, and GHCN). Regions between the two dot-dashed lines show the 68.4% confidence limits [mean ± one standard deviation (SD)] on the CDF plot. Upper and lower limits correspond to mean future precipitation growth rate ± one standard deviation. (**a**), (**b**), (**c**) and (**d**) are for SSP126, SSP245, SSP370, and SSP585 emission scenarios.

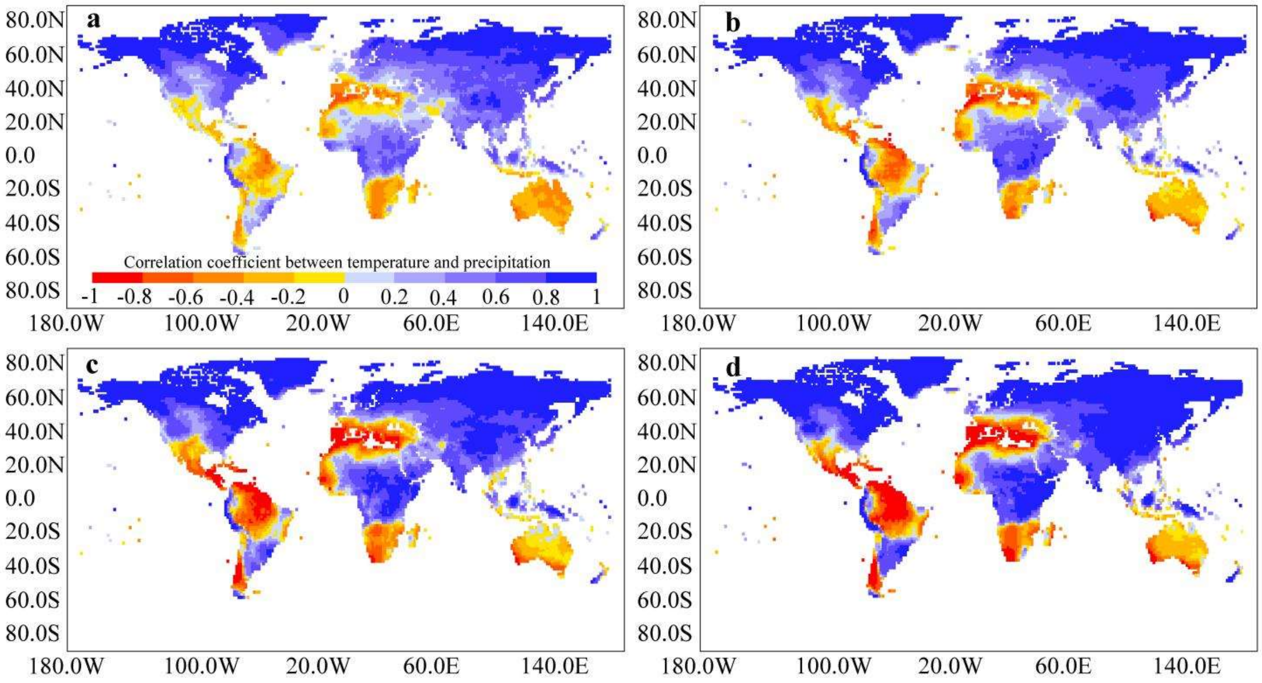

Supplementary Fig. 15. Global distribution of correlation coefficients between precipitation and temperature during 1982–2100 based on CMIP6 ensemble (27 models). (**a**), (**b**), (**c**) and (**d**) show correlation coefficient distributions for SSP126, SSP245, SSP370, and SSP585 emission scenarios.

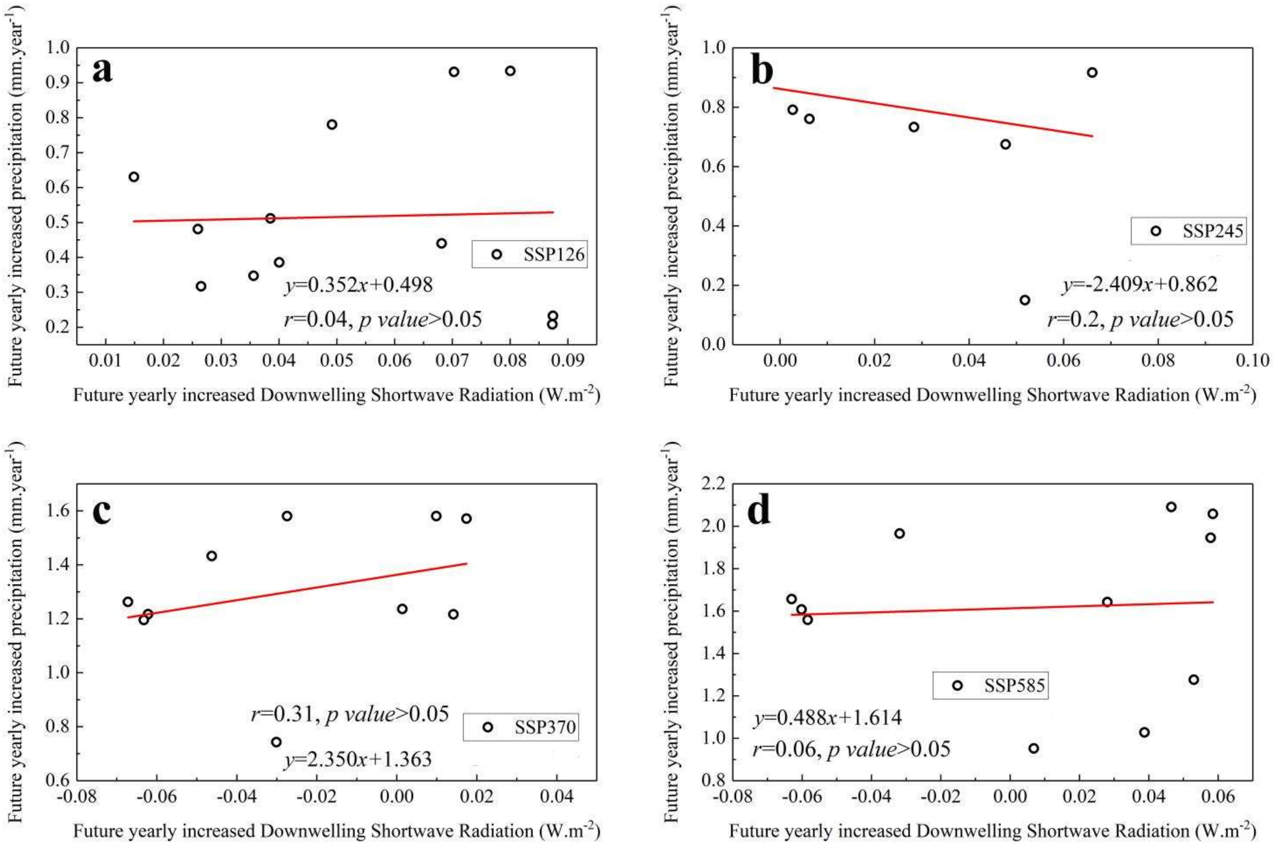

Supplementary Fig. 16. Poor linear relations between future yearly increments of down-welling shortwave radiation (W m^{-2}) and future yearly increments of annual precipitation (mm year^{-1}). (**a**), (**b**), (**c**) and (**d**) are the relations for SSP126, SSP245, SSP370, and SSP585 emission scenarios. Each circle represents the output of one CMIP6 model.

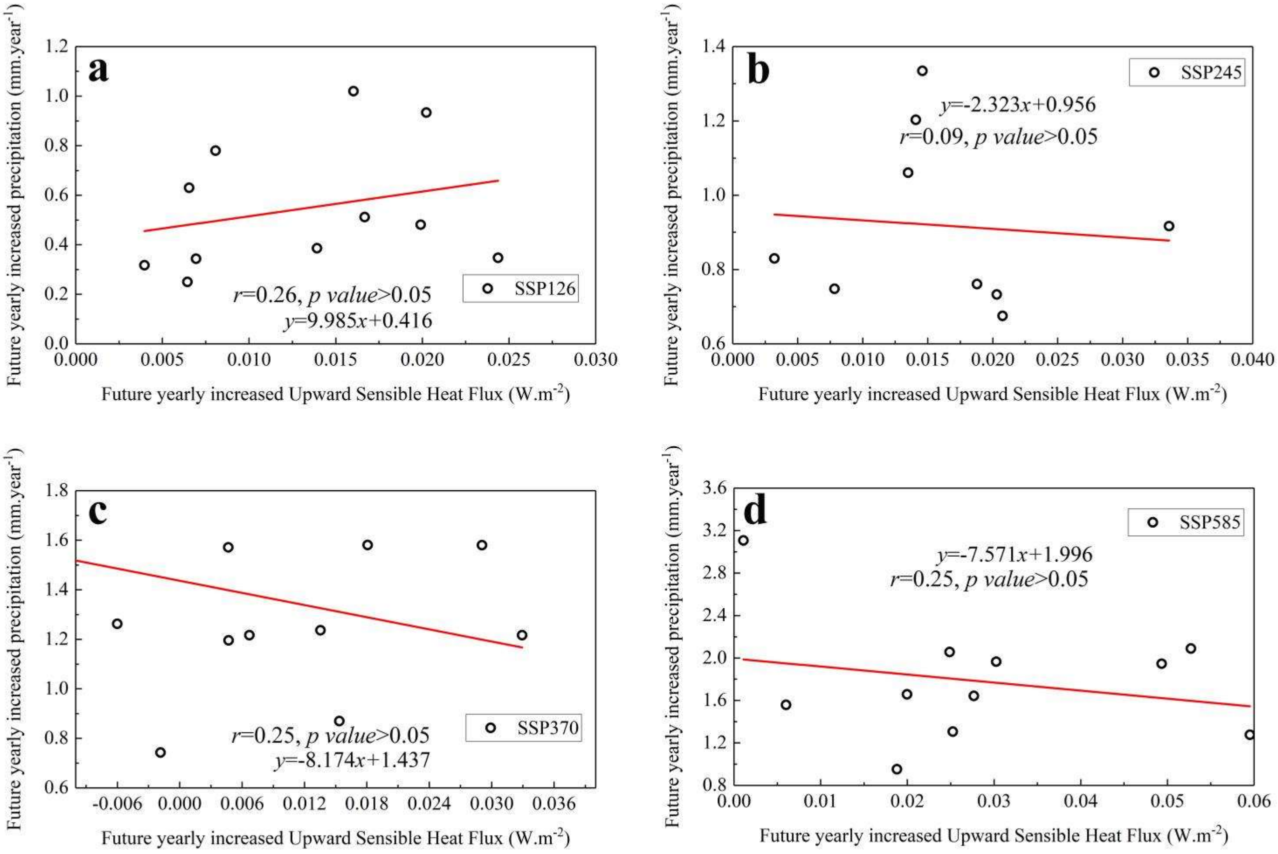

Supplementary Fig. 17. Poor linear relations between future yearly increments of upward sensible heat flux (W m^{-2}) and future yearly increments of annual precipitation (mm year^{-1}). (**a**), (**b**), (**c**) and (**d**) are the relations for SSP126, SSP245, SSP370, and SSP585 emission scenarios. Each circle represents the output of one CMIP6 model.

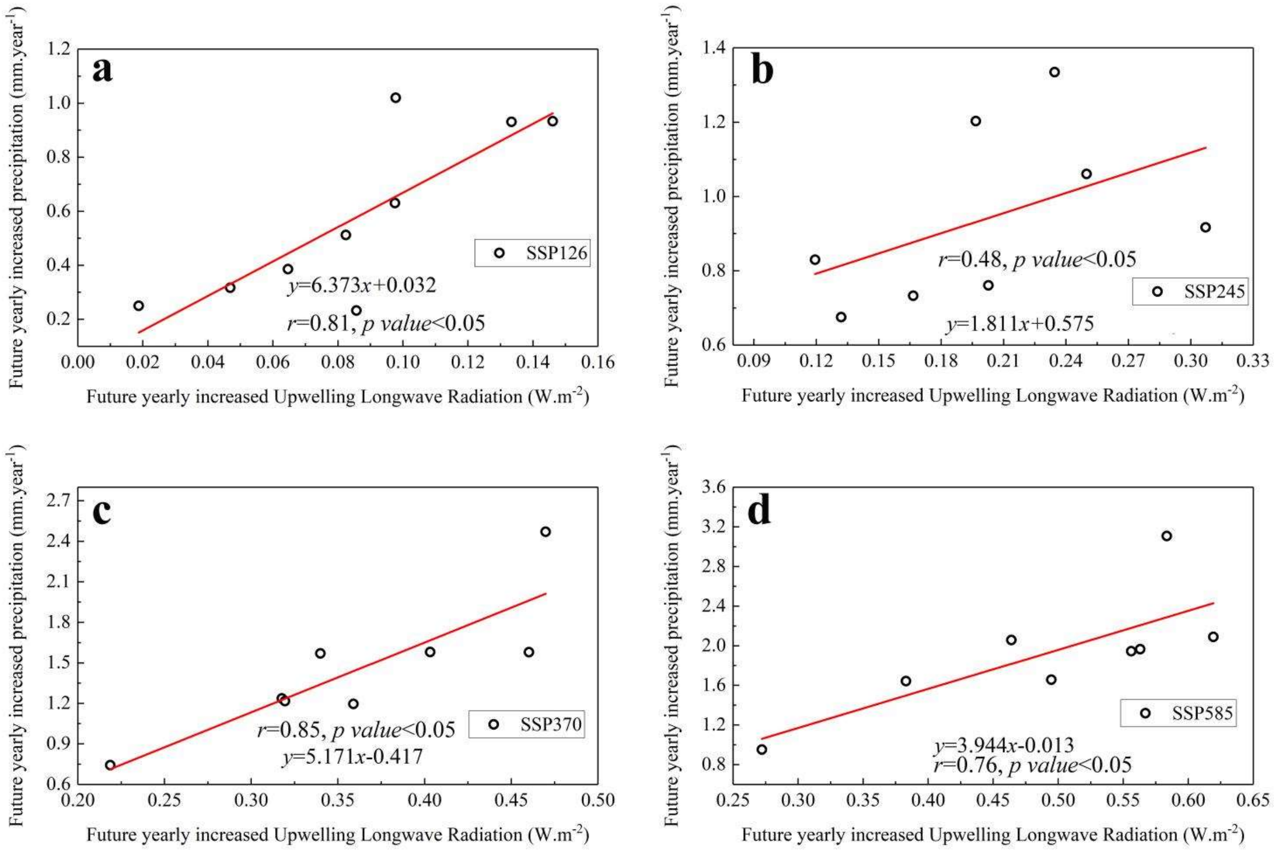

Supplementary Fig. 18. Linear relations between future yearly increments of up-welling longwave radiation (W m^{-2}) and future yearly increments of annual precipitation (mm $year^{-1}$). (**a**), (**b**), (**c**) and (**d**) are the relations for SSP126, SSP245, SSP370, and SSP585 emission scenarios. Each circle represents the output of one CMIP6 model.

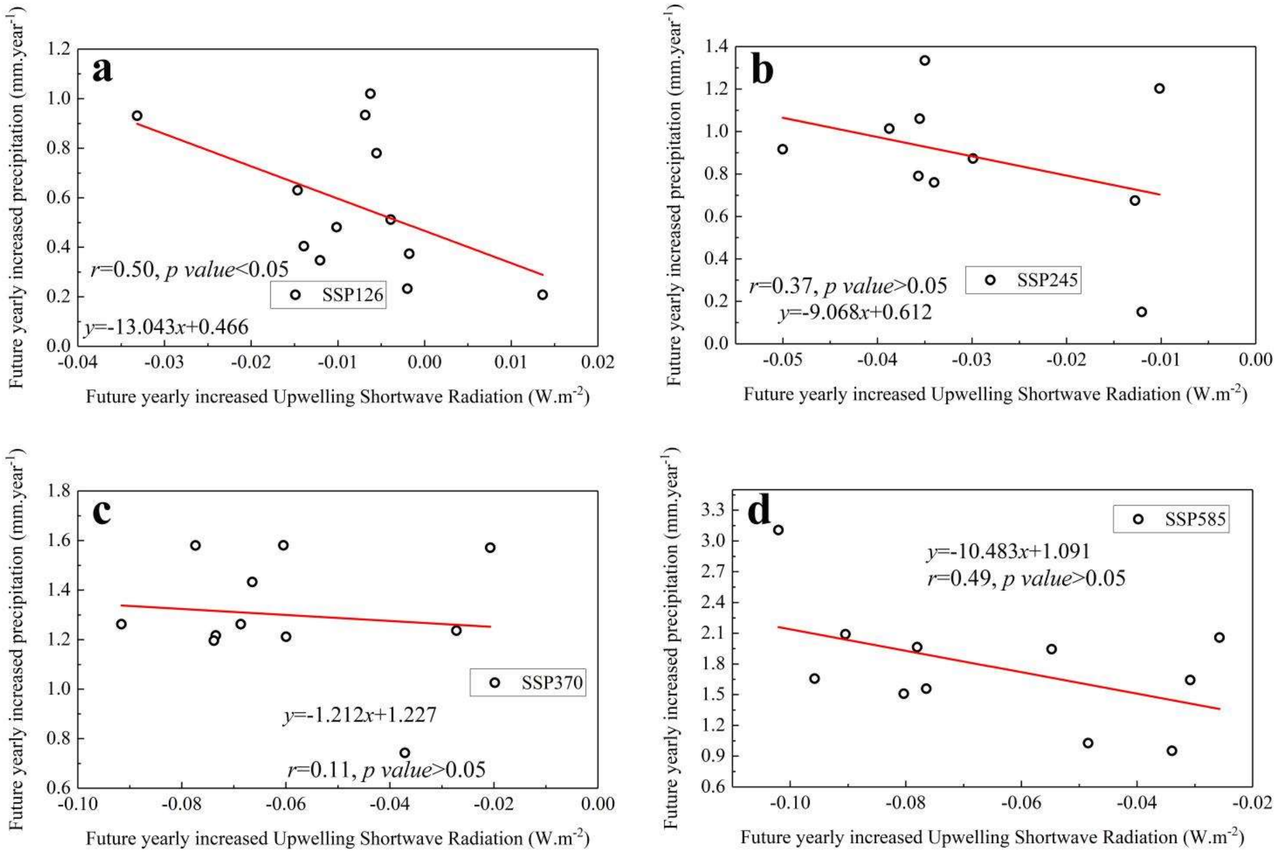

Supplementary Fig. 19. Poor linear relations between future yearly increments of up-welling shortwave radiation (W m^{-2}) and future yearly increments of annual precipitation (mm year^{-1}). (**a**), (**b**), (**c**) and (**d**) are the relations for SSP126, SSP245, SSP370, and SSP585 emission scenarios. Each circle represents the output of one CMIP6 model.

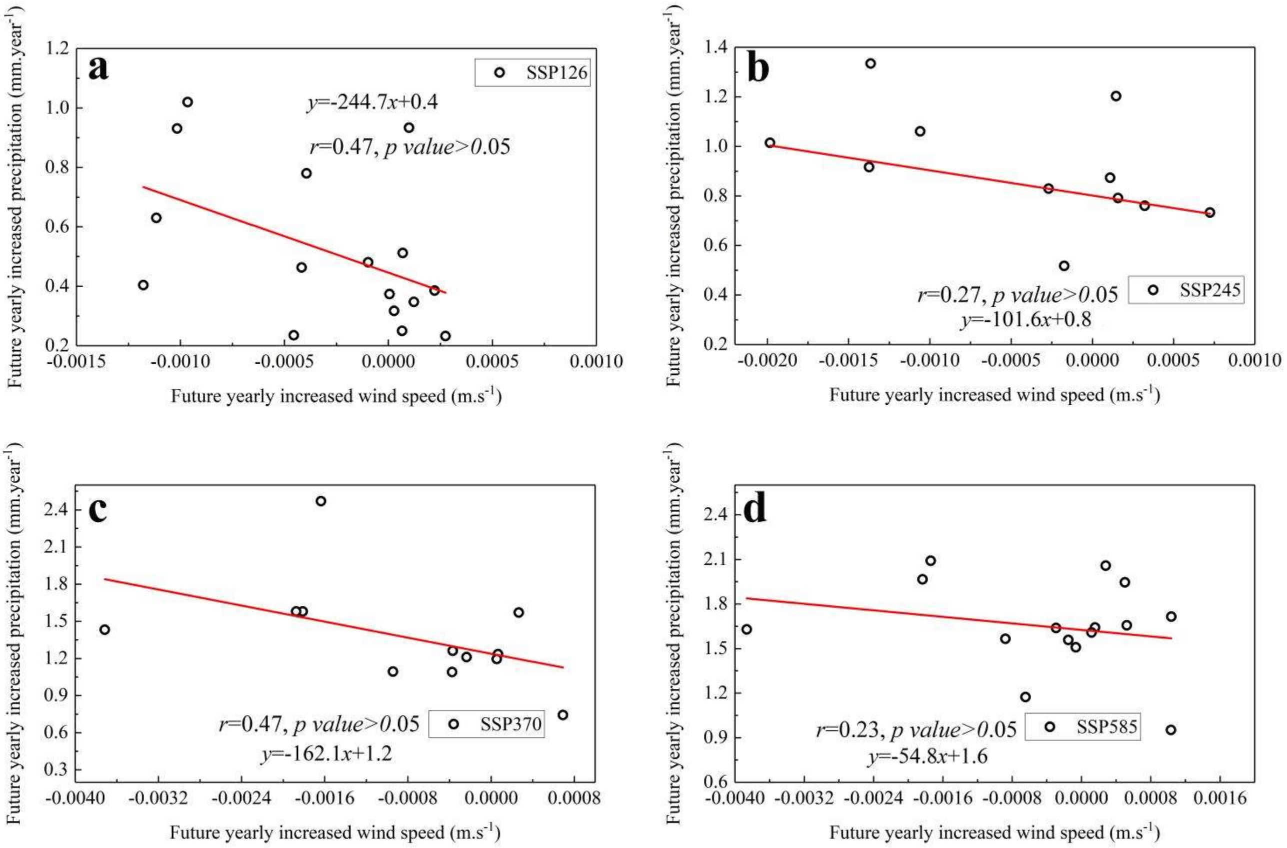

Supplementary Fig. 20. Poor linear relations between future yearly increments of wind speed (m s^{-1}) and future yearly increments of annual precipitation (mm $year^{-1}$). (**a**), (**b**), (**c**) and (**d**) are the relations for SSP126, SSP245, SSP370, and SSP585 emission scenarios. Each circle represents the output of one CMIP6 model.

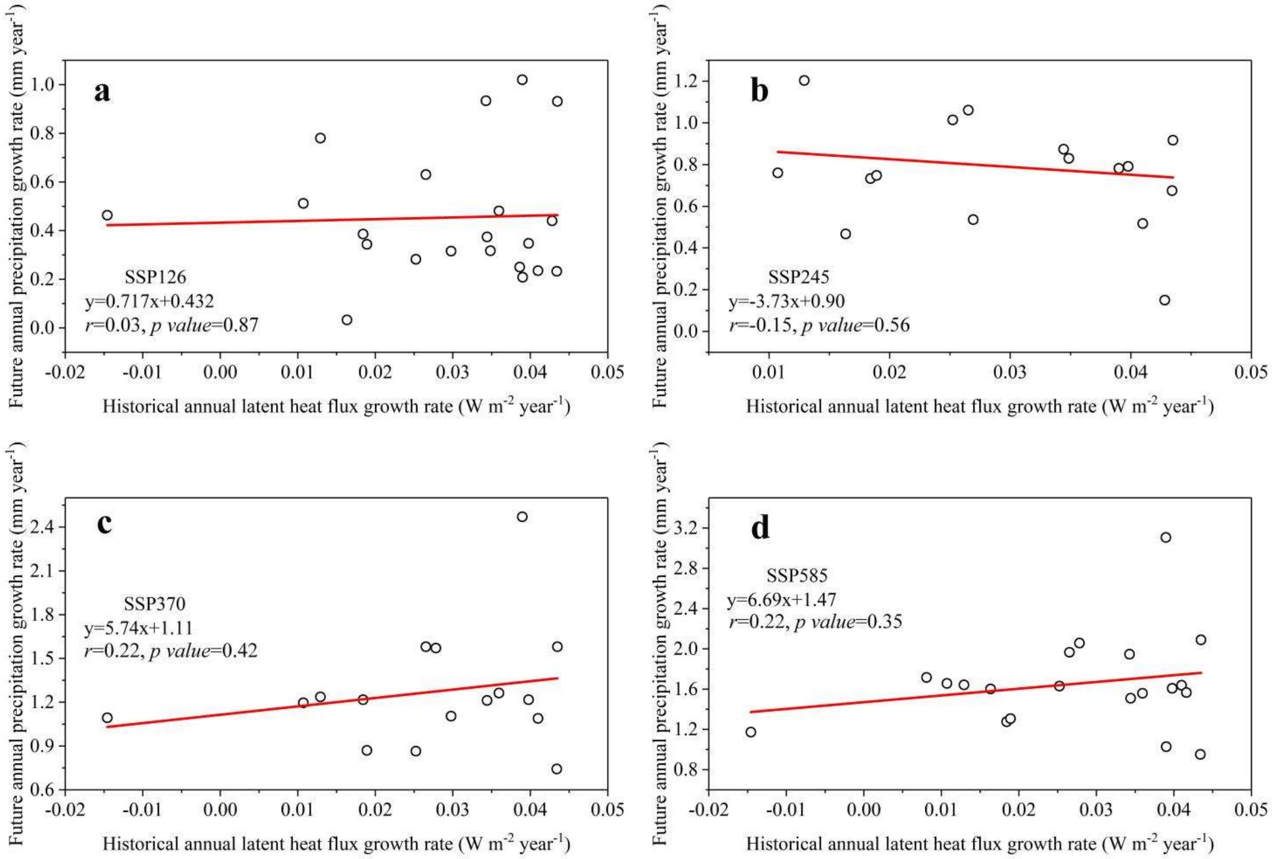

Supplementary Fig. 21. Poor linear relationships between historical annual latent heat flux growth rate (W m^{-2} year^{-1}, 1970–2014) and future annual precipitation growth rate (mm year^{-1}, 2015–2100). (**a**), (**b**), (**c**) and (**d**) are the relations for SSP126, SSP245, SSP370, and SSP585 emission scenarios. Each circle represents one CMIP6 model.

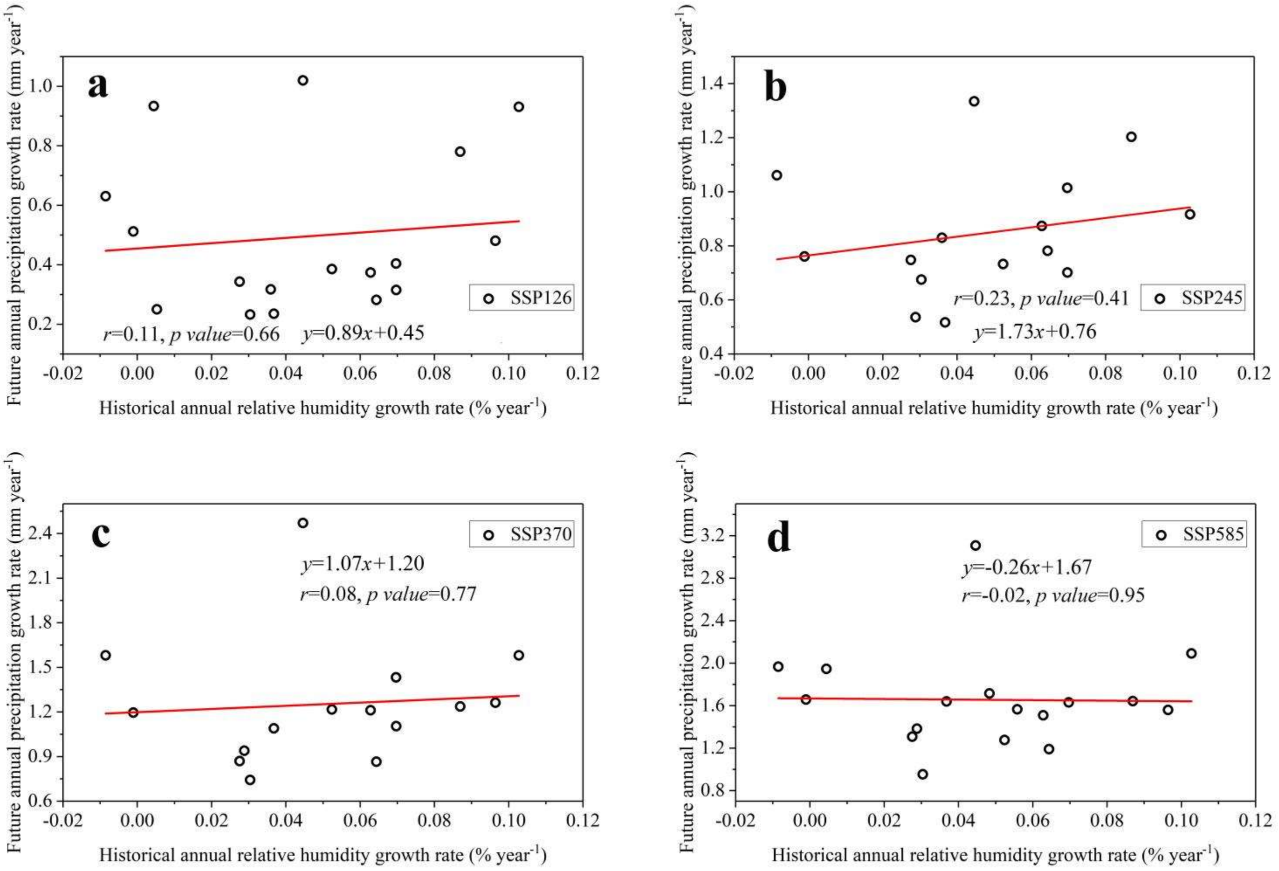

Supplementary Fig. 22. Poor linear relationships between historical annual relative humidity growth rate (1970–2014) and future annual precipitation growth rate (2015–2100). (**a**), (**b**), (**c**) and (**d**) are the relations for SSP126, SSP245, SSP370, and SSP585 emission scenarios. Each circle represents a CMIP6 model

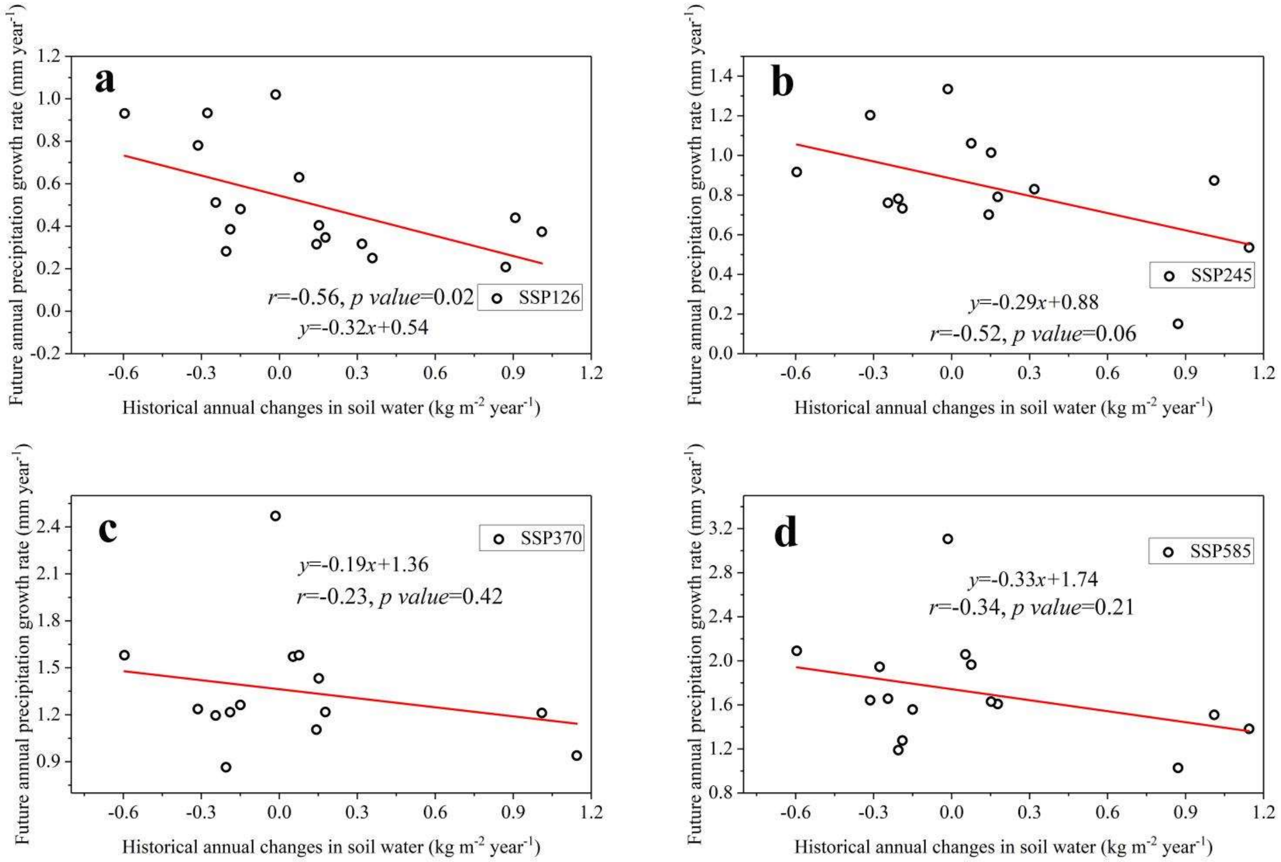

Supplementary Fig. 23. Poor linear relationships between historical annual soil water content decrease rate (1970–2014) and future annual precipitation growth rate (2015–2100). (**a**), (**b**), (**c**) and (**d**) are the relations for SSP126, SSP245, SSP370, and SSP585 emission scenarios. Each circle represents a CMIP6 model

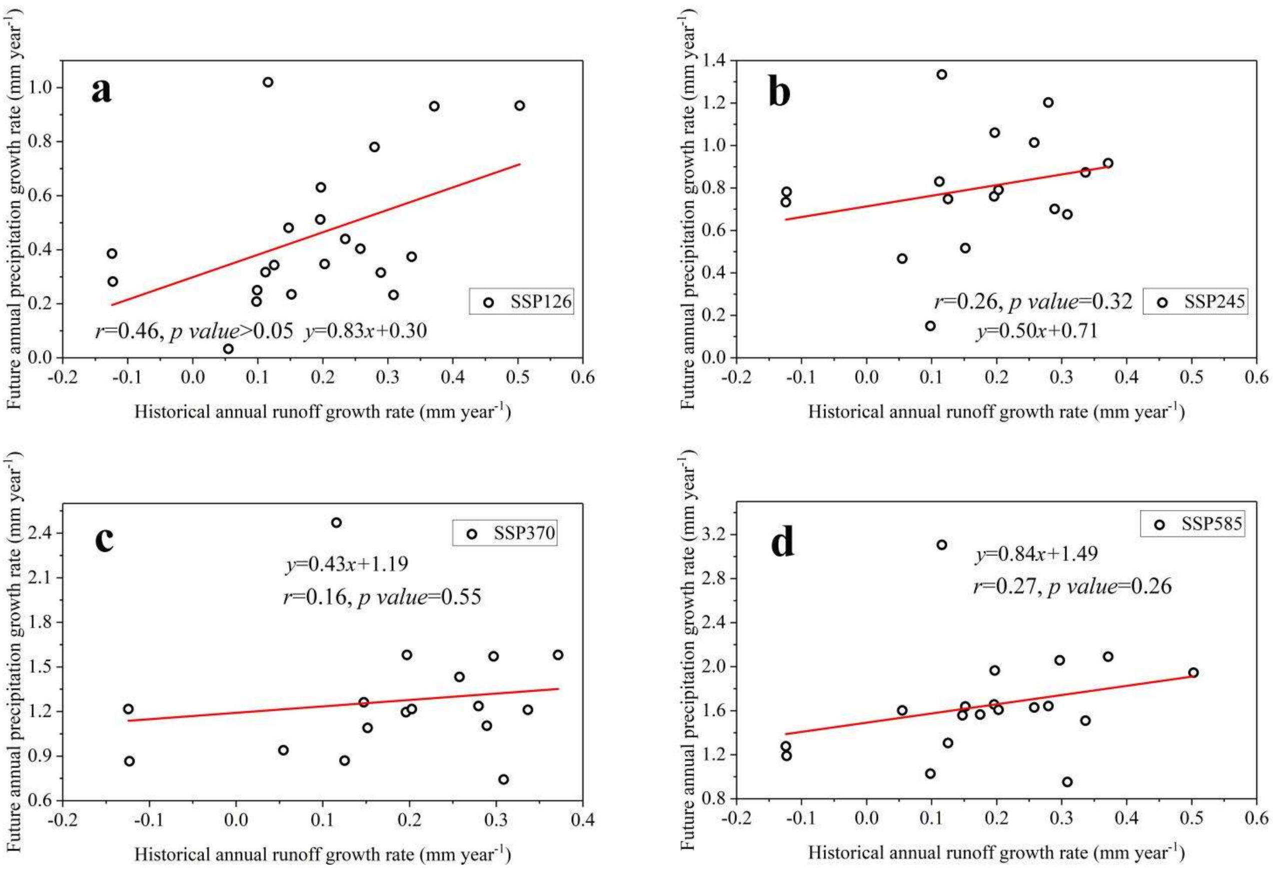

Supplementary Fig. 24. Poor linear relationships between historical annual land surface runoff growth rate (W m^{-2} year^{-1}, 1970–2014) and future annual precipitation growth rate (mm year^{-1}, 2015–2100). (**a**), (**b**), (**c**) and (**d**) are the relations for SSP126, SSP245, SSP370, and SSP585 emission scenarios. Each circle represents a CMIP6 model.

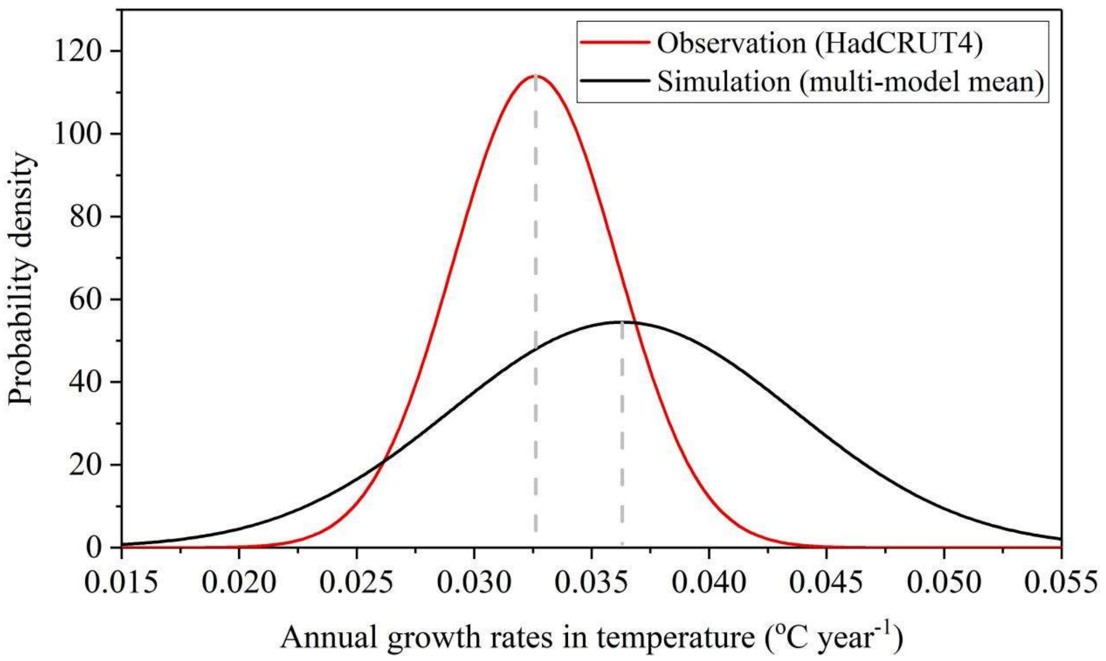

Supplementary Fig. 25. Probability density distributions of annual growth rate in temperature based on observations (red line, 0.0326 ± 0.0035 °C year^{-1}) and CMIP6 multi-model mean values (black line, 0.0363 ± 0.00732 °C year^{-1}).

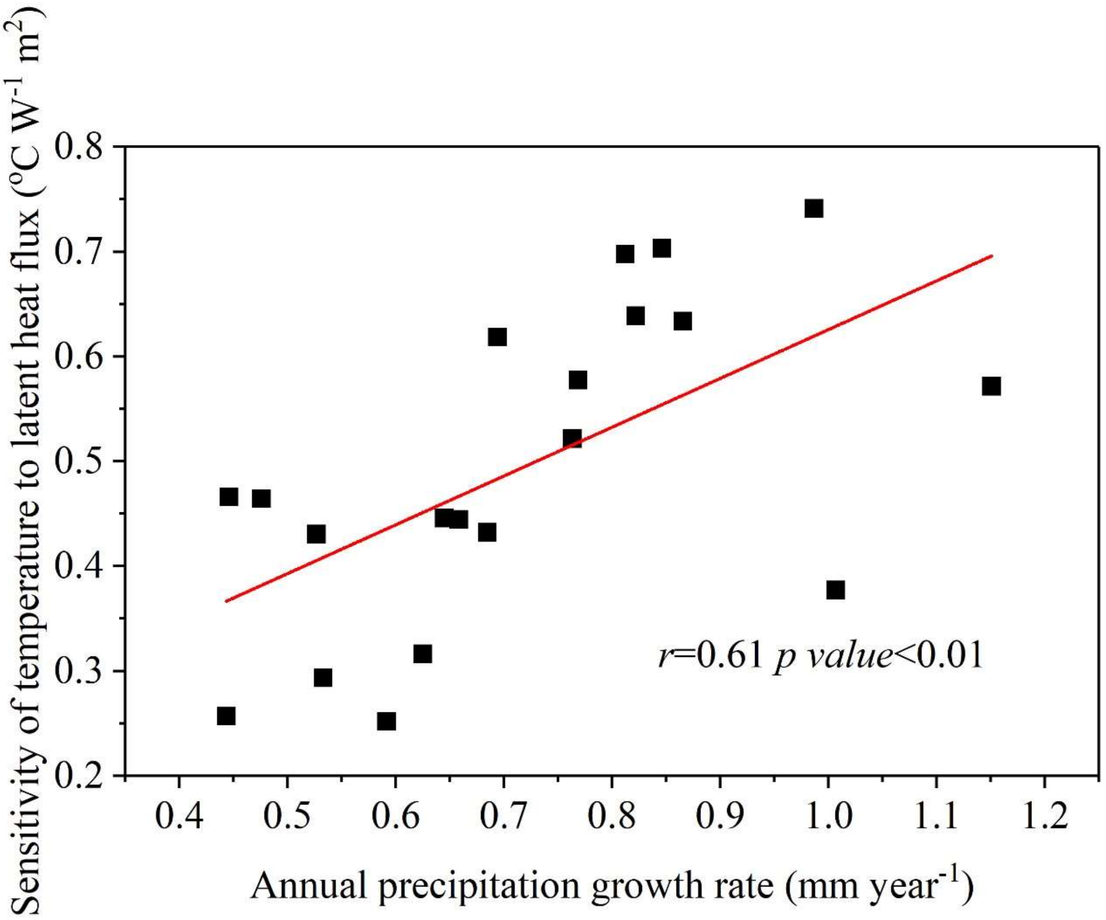

Supplementary Fig. 26. Relationship between annual precipitation growth rate and sensitivity of temperature to latent heat flux during 1970-2014 based on the CMIP6 model projections. One square represents one model.

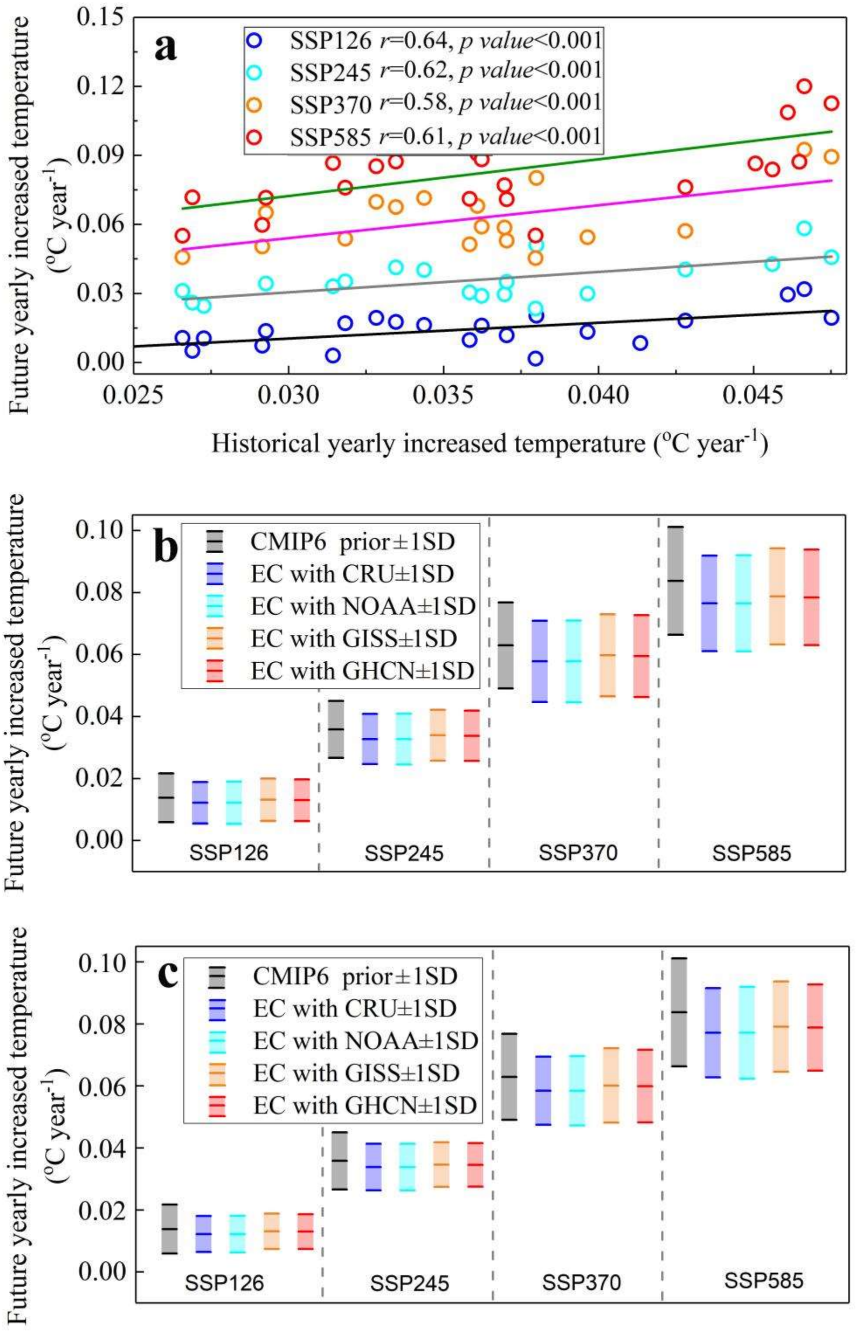

Supplementary Fig. 27. Emergent constraints on future annual temperature growth rate (°C year^{-1}). (**a**) displays emergent constraint relationships between historical annual temperature growth rate and future annual temperature growth rate for SSP126, SSP245, SSP370, and SSP585 emission scenarios. (**b**) and (**c**) present best estimate ± one standard deviation of the original and constrained future annual growth rate of temperature. (**b**) is constrained by observed historical annual temperature growth rates from HadCRUT4, NOAA, GISS, and GHCN data sets (Supplementary Fig. 11a). (**c**) is constrained by the constrained future precipitation growth rate which is derived from the HadCRUT4, NOAA, GISS, and GHCN observational data sets, respectively (also see Figs. 3a and b in the Main text). The constrained results between (**b**) and (**c**) are highly consistent.

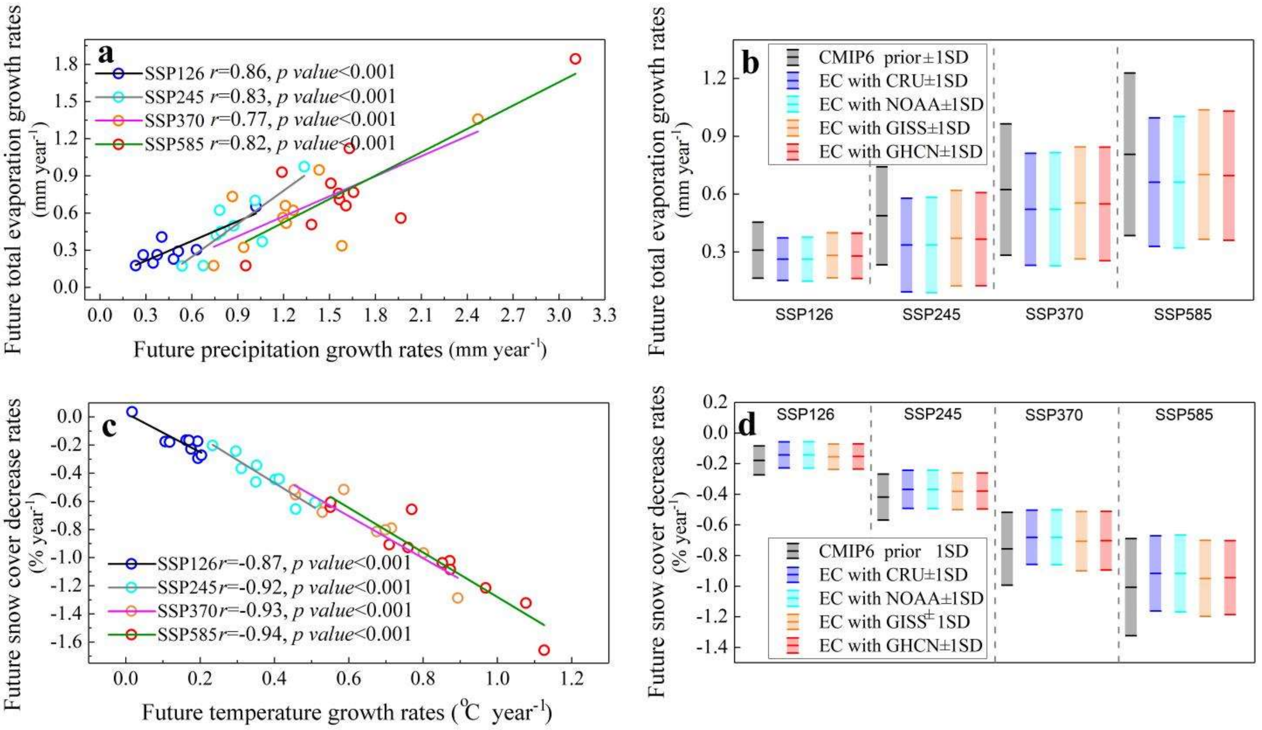

Supplementary Fig. 28. Emergent constraint (EC) on future total evaporation and snow cover fraction changes in Asia after applying the constraint relationship with future precipitation growth rate using the same models involved in developing the original relationships between total evaporation/snow cover fraction and future precipitation growth rates (listed in Supplementary Table 3). (**a**) and **(c)** are the constraint relationships between future annual precipitation and total evaporation growth rates (mm year^{-1}), and between future annual temperature and snow cover fraction (% year^{-1}) growth rates for SSP126, SSP245, SSP370, and SSP585 emission scenarios, with the snow-free regions of Asia excluded; (**b**) and **(d)** are the best estimate ± one standard deviation for future annual total evaporation and for future annual loss in snow cover fraction, when the constrained precipitation is derived from the HadCRUT4, NOAA , GISS, and GHCN observational data sets.

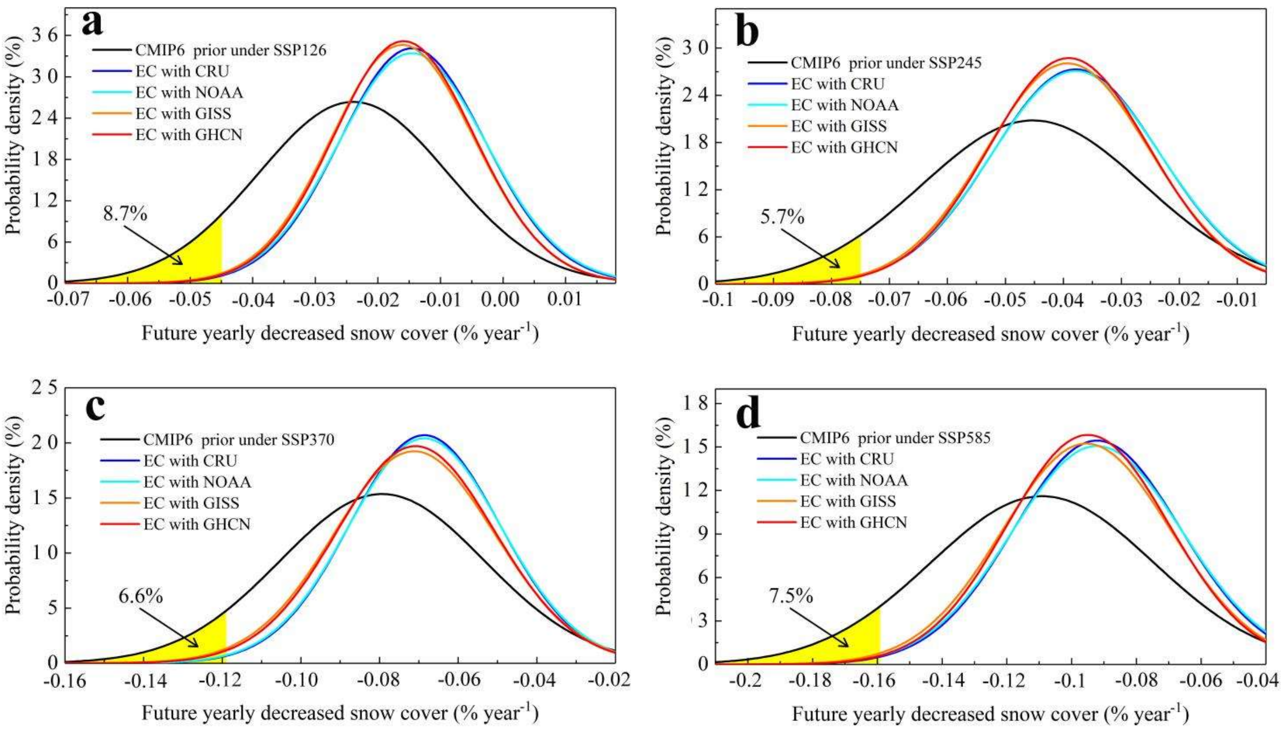

Supplementary Fig. 29. PDFs for the constrained and the unconstrained future annual increments of snow cover fraction (SF) in Asia. (**a**), (**b**), (**c**) and (**d**) are the PDFs for SSP126, SSP245, SSP370, and SSP585 emission scenarios, assuming a Poisson distribution. Yellow shaded areas indicate probability of extreme snow cover losses prior to application of the emergent constraint. Critical snow-cover loss rates obtained by setting the probability to be 0.5% on the PDF curves after the emergent constraint.

Supplementary Tables

Supplementary Table 1. Full names of 27 CMIP6 models and 21 CMIP5 models that supplied data on temperature and precipitation during 1970–2100.

	CMIP6					CMIP5
Number	History	SSP126	SSP245	SSP370	SSP585	History and RCP4.5
1	ACCESS-CM2	ACCESS-ESM1-5	ACCESS-ESM1-5	ACCESS-CM2	ACCESS-CM2	ACCESS1-0
2	ACCESS-ESM1-5	BCC-CSM2-MR	BCC-CSM2-MR	ACCESS-ESM1-5	ACCESS-ESM1-5	CanESM2
3	BCC-CSM2-MR	CanESM5-CanOE	CanESM5-CanOE	BCC-CSM2-MR	BCC-CSM2-MR	CMCC-CM
4	CanESM5-CanOE	CESM2	CESM2	CanESM5-CanOE	CanESM5-CanOE	CMCC-CMS
5	CESM2	CNRM-CM6-1	CNRM-CM6-1	CESM2	CESM2	CSIRO-Mk3-6-0
6	CESM2-WACCM	CNRM-CM6-1-HR	CNRM-CM6-1-HR	CNRM-CM6-1	CESM2-WACCM	CSIRO-Mk3L-1-2
7	CNRM-CM6-1	CNRM-ESM2-1	FGOALS-f3-L	CNRM-CM6-1-HR	CNRM-CM6-1	FGOALS-g2
8	CNRM-CM6-1-HR	FIO-ESM-2-0	FIO-ESM-2-0	CNRM-ESM2-1	CNRM-CM6-1-HR	GFDL-ESM2M
9	CNRM-ESM2-1	GFDL-ESM4	GFDL-CM4	FGOALS-f3-L	CNRM-ESM2-1	GISS-E2-H
10	FGOALS-f3-L	GISS-E2-1-G	GISS-E2-1-G	GFDL-ESM4	FGOALS-f3-L	GISS-E2-H-CC
11	FIO-ESM-2-0	HadGEM3-GC31-LL	INM-CM4-8	GISS-E2-1-G	FIO-ESM-2-0	GISS-E2-R
12	GFDL-CM4	INM-CM4-8	IPSL-CM6A-LR	IPSL-CM6A-LR	GFDL-CM4	GISS-E2-R-CC
13	GFDL-ESM4	INM-CM5-0	MCM-UA-1-0	MCM-UA-1-0	GFDL-ESM4	HadGEM2-AO
14	GISS-E2-1-G	IPSL-CM6A-LR	MIROC6	MIROC6	GISS-E2-1-G	HadGEM2-ES
15	HadGEM3-GC31-LL	MCM-UA-1-0	MIROC-ES2L	MIROC-ES2L	HadGEM3-GC31-LL	inmcm4
16	INM-CM4-8	MIROC6	MPI-ESM1-2-LR	MPI-ESM1-2-LR	IPSL-CM6A-LR	IPSL-CM5A-MR
17	INM-CM5-0	MIROC-ES2L	MRI-ESM2-0	MRI-ESM2-0	KACE-1-0-G	IPSL-CM5B-LR
18	IPSL-CM6A-LR	MPI-ESM1-2-LR	NorESM2-LM	UKESM1-0-LL	MCM-UA-1-0	MIROC5
19	KACE-1-0-G	MRI-ESM2-0	UKESM1-0-LL		MIROC6	MPI-ESM-LR
20	MCM-UA-1-0	NorESM2-LM			MIROC-ES2L	MPI-ESM-MR
21	MIROC6	NorESM2-MM			MRI-ESM2-0	NorESM1-M
22	MIROC-ES2L	UKESM1-0-LL			NorESM2-LM	
23	MPI-ESM1-2-LR				UKESM1-0-LL	
24	MRI-ESM2-0					
25	NorESM2-LM					
26	NorESM2-MM					
27	UKESM1-0-LL					

Supplementary Table 2. Four observed global temperature and precipitation data sets.

Name	Source	Spatial Resolution	Spatial Coverage	Temporal Resolution	Climate variable
HadCRUT4	Met Office Hadley Centre & Climate Research Unit, Univ. of East Anglia, UK	0.5°×0.5°	90N - 90S, 0E - 360E	Monthly	Precipitation and temperature
NOAA	National Oceanic and Atmospheric Administration, USA	5°×5°	87.5S-87.5N, 2.5E - 357.5E	Monthly	Temperature
GISS	Goddard Institute for Space Studies, USA	2°×2°	89.0N - 80.0S, 1.0E - 359.5E	Monthly	Temperature
GHCN	National Climatic Data Center, NC, USA; Arizona State University, USA; Carbon Dioxide Information Analysis Center, USA	5°×5°	87.5S-87.5N, 2.5E - 357.5E	Monthly	Precipitation and temperature
GPCC	Global Precipitation Climatology Centre, USA	0.5°×0.5°	89.75N-89.75S, 0.25E-359.75E	Monthly	Precipitation

Supplementary Table 3. Names of 17 CMIP6 models used to supply data on total evaporation and 14 CMIP6 models used to supply data on snow cover fraction during 2015–2100. Models with names in bold font provided data on both total evaporation and snow cover faction.

No.	Total evaporation				Snow cover fraction			
	**SSP126**	**SSP245**	**SSP370**	**SSP585**	**SSP126**	**SSP245**	**SSP370**	**SSP585**
1	ACCESS-ESM1-5	BCC-CSM2-MR	BCC-CSM2-MR	ACCESS-ESM1-5	**CanESM5-CanOE**	**CanESM5-CanOE**	**CanESM5-CanOE**	**CanESM5-CanOE**
2	BCC-CSM2-MR	**CanESM5-CanOE**	**CanESM5-CanOE**	BCC-CSM2-MR	CESM2	CESM2	CESM2	CESM2
3	**CanESM5-CanOE**	**CNRM-CM6-1**	**CNRM-CM6-1**	**CanESM5-CanOE**	**CNRM-CM6-1**	**CNRM-CM6-1**	**CNRM-CM6-1**	**CESM2-WACCM**
4	**CNRM-CM6-1**	**CNRM-CM6-1-HR**	**CNRM-CM6-1-HR**	**CESM2-WACCM**	**CNRM-CM6-1-HR**	**CNRM-CM6-1-HR**	**CNRM-CM6-1-HR**	**CNRM-CM6-1**
5	**CNRM-CM6-1-HR**	**FGOALS-f3-L**	**CNRM-ESM2-1**	**CNRM-CM6-1**	**CNRM-ESM2-1**	**FGOALS-f3-L**	**CNRM-ESM2-1**	**CNRM-CM6-1-HR**
6	**CNRM-ESM2-1**	FIO-ESM-2-0	**FGOALS-f3-L**	**CNRM-CM6-1-HR**	**GISS-E2-1-G**	**GISS-E2-1-G**	**FGOALS-f3-L**	**CNRM-ESM2-1**
7	FIO-ESM-2-0	**GISS-E2-1-G**	**GISS-E2-1-G**	**CNRM-ESM2-1**	HadGEM3-GC31-LL	**IPSL-CM6A-LR**	**GISS-E2-1-G**	**FGOALS-f3-L**
8	**GISS-E2-1-G**	INM-CM4-8	**IPSL-CM6A-LR**	**FGOALS-f3-L**	**IPSL-CM6A-LR**	**MIROC6**	**IPSL-CM6A-LR**	**GISS-E2-1-G**
9	INM-CM4-8	**IPSL-CM6A-LR**	MCM-UA-1-0	FIO-ESM-2-0	**MIROC6**	**MIROC-ES2L**	**MIROC6**	HadGEM3-GC31-LL
10	INM-CM5-0	MCM-UA-1-0	**MIROC6**	**GISS-E2-1-G**	**MIROC-ES2L**	**MRI-ESM2-0**	**MIROC-ES2L**	**IPSL-CM6A-LR**
11	**IPSL-CM6A-LR**	**MIROC6**	**MIROC-ES2L**	**IPSL-CM6A-LR**	**MRI-ESM2-0**	UKESM1-0-LL	**MRI-ESM2-0**	**MIROC6**
12	MCM-UA-1-0	**MIROC-ES2L**	**MRI-ESM2-0**	KACE-1-0-G	UKESM1-0-LL		UKESM1-0-LL	**MIROC-ES2L**
13	**MIROC6**	**MRI-ESM2-0**		MCM-UA-1-0				**MRI-ESM2-0**
14	**MIROC-ES2L**	NorESM2-LM		**MIROC6**				UKESM1-0-LL
15	**MRI-ESM2-0**			**MIROC-ES2L**				
16	NorESM2-LM			**MRI-ESM2-0**				
17				NorESM2-LM				

Supplementary Table 4. Names of CMIP6 models that provided data on Downwelling Longwave Radiation and Downward Sensible Heat Flux during 1970–2100.

No.	Downwelling Longwave Radiation					Downward Sensible Heat Flux				
	History	SSP126	SSP245	SSP370	SSP585	History	SSP126	SSP245	SSP370	SSP585
1	CNRM-CM6-1	HadGEM3-GC31-LL	INM-CM5-0	INM-CM4-8	HadGEM3-GC31-LL	ACCESS-CM2	ACCESS-CM2	ACCESS-CM2	ACCESS-CM2	ACCESS-ESM1-5
2	CNRM-ESM2-1	HadGEM3-GC31-MM	IPSL-CM6A-LR	INM-CM5-0	HadGEM3-GC31-MM	ACCESS-ESM1-5	ACCESS-ESM1-5	ACCESS-ESM1-5	ACCESS-ESM1-5	CESM2-WACCM
3	HadGEM3-GC31-LL	INM-CM4-8	MIROC-ES2L	IPSL-CM6A-LR	INM-CM4-8	CESM2	CESM2-WACCM	CESM2-WACCM	CESM2-WACCM	FGOALS-f3-L
4	INM-CM4-8	IPSL-CM6A-LR	UKESM1-0-LL	MIROC-ES2L	INM-CM5-0	MRI-ESM2-0	FGOALS-f3-L	FGOALS-f3-L	INM-CM4-8	INM-CM4-8
5	MIROC-ES2L	MIROC-ES2L		UKESM1-0-LL	IPSL-CM6A-LR		INM-CM4-8	INM-CM4-8	INM-CM5-0	INM-CM5-0
6		UKESM1-0-LL			MIROC-ES2L		INM-CM5-0	INM-CM5-0	MRI-ESM2-0	MRI-ESM2-0
7					UKESM1-0-LL		MRI-ESM2-0	MRI-ESM2-0		

Supplementary Table 5. Names of CMIP6 models that provided data on Downwelling Shortwave Radiation and Surface Upward Sensible Heat Flux during 1970–2100.

No.	Downwelling Shortwave Radiation					Surface Upward Sensible Heat Flux				
	History	SSP126	SSP245	SSP370	SSP585	History	SSP126	SSP245	SSP370	SSP585
1	ACCESS-CM2	ACCESS-CM2	ACCESS-CM2	ACCESS-CM2	ACCESS-CM2	ACCESS-CM2	ACCESS-CM2	ACCESS-CM2	ACCESS-CM2	ACCESS-CM2
2	ACCESS-ESM1-5	ACCESS-ESM1-5	ACCESS-ESM1-5	ACCESS-ESM1-5	ACCESS-ESM1-5	ACCESS-ESM1-5	ACCESS-ESM1-5	ACCESS-ESM1-5	ACCESS-ESM1-5	ACCESS-ESM1-5
3	BCC-ESM1	BCC-CSM2-MR	BCC-CSM2-MR	BCC-CSM2-MR	BCC-CSM2-MR	BCC-CSM2-MR	BCC-CSM2-MR	BCC-CSM2-MR	BCC-CSM2-MR	BCC-CSM2-MR
4	CNRM-CM6-1	CNRM-CM6-1	CNRM-CM6-1	CNRM-CM6-1	CNRM-CM6-1	CanESM5-CanOE	CanESM5	CanESM5	CanESM5	CanESM5
5	CNRM-CM6-1-HR	CNRM-CM6-1-HR	CNRM-CM6-1-HR	CNRM-CM6-1-HR	CNRM-CM6-1-HR	CNRM-CM6-1	CanESM5-CanOE	CanESM5-CanOE	CanESM5-CanOE	CanESM5-CanOE
6	CNRM-ESM2-1	CNRM-ESM2-1	CNRM-ESM2-1	CNRM-ESM2-1	CNRM-ESM2-1	CNRM-CM6-1-HR	CNRM-CM6-1	CNRM-CM6-1	CNRM-CM6-1	CNRM-CM6-1
7	GISS-E2-1-G	GISS-E2-1-G	GISS-E2-1-G	GISS-E2-1-G	GISS-E2-1-G	GISS-E2-1-G	CNRM-CM6-1-HR	CNRM-ESM2-1	CNRM-CM6-1-HR	CNRM-ESM2-1
8	GISS-E2-1-H	HadGEM3-GC31-LL	HadGEM3-GC31-LL	INM-CM4-8	HadGEM3-GC31-LL	GISS-E2-1-H	CNRM-ESM2-1	GISS-E2-1-G	CNRM-ESM2-1	GISS-E2-1-G
9	HadGEM3-GC31-LL	HadGEM3-GC31-MM	IPSL-CM6A-LR	IPSL-CM6A-LR	INM-CM4-8	HadGEM3-GC31-LL	HadGEM3-GC31-LL	HadGEM3-GC31-LL	GISS-E2-1-G	HadGEM3-GC31-LL
10	HadGEM3-GC31-MM	INM-CM4-8	NorESM2-LM	MIROC-ES2L	IPSL-CM6A-LR	INM-CM4-8	HadGEM3-GC31-MM	INM-CM4-8	INM-CM4-8	HadGEM3-GC31-MM
11	IPSL-CM6A-LR	IPSL-CM6A-LR	NorESM2-MM	NorESM2-LM	NorESM2-LM	INM-CM5-0	INM-CM4-8	INM-CM5-0	INM-CM5-0	INM-CM4-8
12	MIROC-ES2L	NorESM2-LM	UKESM1-0-LL	NorESM2-MM	NorESM2-MM	IPSL-CM6A-LR	INM-CM5-0	IPSL-CM6A-LR	IPSL-CM6A-LR	INM-CM5-0
13		NorESM2-MM		UKESM1-0-LL	UKESM1-0-LL	MCM-UA-1-0	IPSL-CM6A-LR	MCM-UA-1-0	MCM-UA-1-0	IPSL-CM6A-LR
14		UKESM1-0-LL				MPI-ESM-1-2-HAM	MCM-UA-1-0	MPI-ESM1-2-HR	UKESM1-0-LL	MCM-UA-1-0
15						UKESM1-0-LL	MPI-ESM1-2-HR	UKESM1-0-LL		UKESM1-0-LL
16							UKESM1-0-LL			

Supplementary Table 6. Names of CMIP6 models that provided data on Surface Upwelling Longwave Radiation and Surface Upwelling Shortwave Radiation during 1970–2100.

No.	Surface Upwelling Longwave Radiation					Surface Upwelling Shortwave Radiation				
	History	SSP126	SSP245	SSP370	SSP585	History	SSP126	SSP245	SSP370	SSP585
1	ACCESS-CM2	ACCESS-CM2	ACCESS-CM2	ACCESS-CM2	ACCESS-CM2	ACCESS-CM2	ACCESS-CM2	ACCESS-CM2	ACCESS-CM2	ACCESS-CM2
2	BCC-CSM2-MR	BCC-CSM2-MR	ACCESS-ESM1-5	ACCESS-ESM1-5	ACCESS-ESM1-5	ACCESS-ESM1-5	ACCESS-ESM1-5	ACCESS-ESM1-5	ACCESS-ESM1-5	ACCESS-ESM1-5
3	BCC-ESM1	CNRM-CM6-1	BCC-CSM2-MR	BCC-CSM2-MR	CNRM-CM6-1	CNRM-CM6-1-HR	CNRM-CM6-1-HR	CNRM-CM6-1-HR	CNRM-CM6-1-HR	CNRM-CM6-1
4	CNRM-CM6-1-HR	CanESM5-CanOE	CNRM-CM6-1	CNRM-CM6-1	CanESM5-CanOE	CNRM-CM6-1	CNRM-CM6-1	CNRM-CM6-1	CNRM-CM6-1	CNRM-ESM2-1
5	CNRM-CM6-1	CanESM5	CanESM5-CanOE	CanESM5-CanOE	CanESM5	CNRM-ESM2-1	CNRM-ESM2-1	CNRM-ESM2-1	CNRM-ESM2-1	CanESM5-CanOE
6	CanESM5-CanOE	GISS-E2-1-G	CanESM5	CanESM5	GISS-E2-1-G	CanESM5	CanESM5-CanOE	CanESM5-CanOE	CanESM5-CanOE	CanESM5
7	CanESM5	HadGEM3-GC31-LL	GISS-E2-1-G	GISS-E2-1-G	HadGEM3-GC31-LL	HadGEM3-GC31-LL	CanESM5	CanESM5	CanESM5	GISS-E2-1-G
8	GISS-E2-1-G	HadGEM3-GC31-MM	HadGEM3-GC31-LL	INM-CM4-8	HadGEM3-GC31-MM	HadGEM3-GC31-MM	GISS-E2-1-G	GISS-E2-1-G	GISS-E2-1-G	HadGEM3-GC31-LL
9	HadGEM3-GC31-LL	INM-CM4-8	INM-CM4-8	INM-CM5-0	INM-CM4-8	IPSL-CM6A-LR	HadGEM3-GC31-LL	HadGEM3-GC31-LL	IPSL-CM6A-LR	HadGEM3-GC31-MM
10	HadGEM3-GC31-MM	INM-CM5-0	INM-CM5-0	IPSL-CM6A-LR	INM-CM5-0	MIROC-ES2L	HadGEM3-GC31-MM	IPSL-CM6A-LR	MIROC-ES2L	IPSL-CM6A-LR
11	INM-CM4-8	IPSL-CM6A-LR	IPSL-CM6A-LR	UKESM1-0-LL	IPSL-CM6A-LR	UKESM1-0-LL	IPSL-CM6A-LR	MIROC-ES2L	MIROC6	MIROC6
12	INM-CM5-0	UKESM1-0-LL	UKESM1-0-LL		UKESM1-0-LL		MIROC-ES2L	MIROC6	NorESM2-LM	NorESM2-LM
13	IPSL-CM6A-LR						MIROC6	NorESM2-LM	UKESM1-0-LL	UKESM1-0-LL
14	MPI-ESM-1-2-HAM						NorESM2-LM	UKESM1-0-LL		
15	UKESM1-0-LL						UKESM1-0-LL			

Supplementary Table 7. Names of CMIP6 models used for wind during 1970–2100.

No.	Wind				
	History	SSP126	SSP245	SSP370	SSP585
1	ACCESS-CM2	ACCESS-CM2	ACCESS-CM2	ACCESS-CM2	ACCESS-CM2
2	ACCESS-ESM1-5	ACCESS-ESM1-5	ACCESS-ESM1-5	ACCESS-ESM1-5	ACCESS-ESM1-5
3	CAMS-CSM1-0	BCC-CSM2-MR	BCC-CSM2-MR	CESM2-WACCM	CESM2-WACCM
4	CESM2-WACCM	CanESM5	CESM2-WACCM	CESM2	CESM2
5	CESM2	CanESM5-CanOE	CESM2	CNRM-CM6-1	CNRM-CM6-1-HR
6	CNRM-CM6-1-HR	CESM2	CNRM-CM6-1-HR	CNRM-ESM2-1	CNRM-CM6-1
7	CNRM-CM6-1	CESM2-WACCM	CNRM-CM6-1	CanESM5-CanOE	CNRM-ESM2-1
8	CNRM-ESM2-1	CNRM-CM6-1	CNRM-ESM2-1	CanESM5	CanESM5
9	CanESM5-CanOE	CNRM-CM6-1-HR	CanESM5-CanOE	GFDL-ESM4	GFDL-ESM4
10	GFDL-ESM4	CNRM-ESM2-1	CanESM5	GISS-E2-1-G	GISS-E2-1-G
11	GISS-E2-1-G	GFDL-ESM4	GFDL-ESM4	INM-CM4-8	HadGEM3-GC31-LL
12	HadGEM3-GC31-LL	GISS-E2-1-G	HadGEM3-GC31-LL	INM-CM5-0	HadGEM3-GC31-MM
13	HadGEM3-GC31-MM	HadGEM3-GC31-LL	INM-CM4-8	IPSL-CM6A-LR	INM-CM4-8
14	INM-CM4-8	HadGEM3-GC31-MM	INM-CM5-0	KACE-1-0-G	INM-CM5-0
15	INM-CM5-0	INM-CM4-8	IPSL-CM6A-LR	MIROC-ES2L	IPSL-CM6A-LR
16	IPSL-CM6A-LR	INM-CM5-0	KACE-1-0-G	MIROC-ES2L	KACE-1-0-G
17	KACE-1-0-G	IPSL-CM6A-LR	MIROC-ES2L	MIROC6	MIROC-ES2L
18	MIROC-ES2L	KACE-1-0-G	MIROC6	UKESM1-0-LL	MIROC6
19	NorESM2-LM	MIROC6	NorESM2-LM		UKESM1-0-LL
20	UKESM1-0-LL	MIROC-ES2L	UKESM1-0-LL		
21		UKESM1-0-LL			

Supplementary Table 8. Observed yearly increase in temperature ± one standard deviation from the four data sets during 1970 – 2014 and predicted yearly increase in annual precipitation (Δ*P*) ± one standard deviation based on CMIP6 projections before and after emergent constraint during 2015 – 2100. Note: Overestimated Δ*P* = (Unconstrained Δ*P* - Constrained Δ*P*)/Unconstrained Δ*P*; Reduced uncertainty in Δ*P* = (Unconstrained standard deviation of Δ*P* - Constrained standard deviation of Δ*P*)/Unconstrained standard deviation of Δ*P*

	**Observed yearly increase in temperature** (°C $year^{-1}$)	Emission Scenario	Future yearly increase in annual precipitation before emergent constraint (mm $year^{-1}$)		Future yearly increase in annual precipitation after emergent constraint (mm $year^{-1}$)		Overestimated future yearly increase in annual precipitation by original CMIP6 outputs (%)	Reduced uncertainty in future yearly increase in annual precipitation after emergent constraint (%)
			Mean value	One standard deviation	Mean value	One standard deviation		
HadCRUT4	0.0326 ± 0.0035	**SSP126**	0.451	0.258	0.388	0.178	14.0%	31.0%
		**SSP245**	0.799	0.277	0.699	0.222	12.5%	19.9%
		**SSP370**	1.260	0.389	1.102	0.332	12.5%	14.7%
		**SSP585**	1.622	0.444	1.418	0.350	12.6%	21.2%
NOAA	0.0326 ± 0.0036	**SSP126**	0.451	0.258	0.388	0.188	14.0%	27.1%
		**SSP245**	0.799	0.277	0.699	0.229	12.5%	17.3%
		**SSP370**	1.260	0.389	1.102	0.342	12.5%	12.1%
		**SSP585**	1.622	0.444	1.418	0.368	12.6%	17.1%
GISS	0.0340 ± 0.0038	**SSP126**	0.451	0.258	0.424	0.184	6.0%	28.7%
		**SSP245**	0.799	0.277	0.739	0.229	7.5%	17.3%
		**SSP370**	1.260	0.389	1.162	0.333	7.8%	14.4%
		**SSP585**	1.622	0.444	1.480	0.361	8.8%	18.7%
GHCN	0.0338 ± 0.0036	**SSP126**	0.451	0.258	0.419	0.189	7.1%	26.7%
		**SSP245**	0.799	0.277	0.734	0.223	8.1%	19.5%
		**SSP370**	1.260	0.389	1.154	0.334	8.4%	14.1%
		**SSP585**	1.622	0.444	1.471	0.352	9.3%	20.7%

Supplementary Table 9. Kolmogorov-Smirnov statistics of probability density functions (PDFs) for future precipitation growth rates before and after emergent constraint and the critical value at the 95% confidence level. If the Kolmogorov-Smirnov statistics are higher than the critical value at the 95% confidence level, the shifts of the PDF are significant at the 95% confidence level before and after emergent constraint for all the data sets.

Emission Scenario	Constraining by different observational data sets	Kolmogorov-Smirnov statistics (D_n)	Critical value at the 95% confidence level ($D_{crit,0.01}$)
SSP126	**HadCRUT4**	0.140	0.061
	**NOAA**	0.134	0.061
	**GISS**	0.120	0.061
	**GHCN**	0.134	0.061
SSP245	**HadCRUT4**	0.091	0.061
	**NOAA**	0.089	0.061
	**GISS**	0.073	0.061
	**GHCN**	0.074	0.061
SSP370	**HadCRUT4**	0.064	0.061
	**NOAA**	0.064	0.061
	**GISS**	0.062	0.061
	**GHCN**	0.063	0.061
SSP585	**HadCRUT4**	0.076	0.061
	**NOAA**	0.074	0.061
	**GISS**	0.068	0.061
	**GHCN**	0.070	0.061

Supplementary Table 10. Constraint on future yearly increase in temperature using constrained future yearly increase in annual precipitation.

	Emission Scenario	Constrained future yearly increase in annual precipitation ± one standard deviation (mm $year^{-1}$)	Future yearly increase in temperature before constraint (^oC $year^{-1}$)		Future yearly increase in temperature after constraint (^oC $year^{-1}$)		Overestimated future yearly increase in temperature by original CMIP6 outputs (%)	Reduced uncertainty in future yearly increase in temperature after constraint (%)
			Mean value	One standard deviation	Mean value	One standard deviation		
HadCRUT4	**SSP126**	0.388 ± 0.178	0.0138	0.0079	0.0122	0.0058	11.6%	26.6%
	**SSP245**	0.699 ± 0.222	0.0358	0.0092	0.0338	0.0075	5.6%	18.5%
	**SSP370**	1.102 ± 0.332	0.0629	0.0139	0.0584	0.0110	7.2%	20.9%
	**SSP585**	1.418 ± 0.350	0.0837	0.0174	0.0771	0.0144	7.9%	17.2%
NOAA	**SSP126**	0.388 ± 0.188	0.0138	0.0079	0.0122	0.0059	11.6%	25.3%
	**SSP245**	0.699 ± 0.229	0.0358	0.0092	0.0338	0.0075	5.6%	18.5%
	**SSP370**	1.102 ± 0.342	0.0629	0.0139	0.0584	0.0112	7.2%	19.4%
	**SSP585**	1.418 ± 0.368	0.0837	0.0174	0.0771	0.0148	7.9%	14.9%
GISS	**SSP126**	0.424 ± 0.184	0.0138	0.0079	0.0131	0.0057	5.1%	27.8%
	**SSP245**	0.739 ± 0.229	0.0358	0.0092	0.0346	0.0072	3.4%	21.7%
	**SSP370**	1.162 ± 0.333	0.0629	0.0139	0.0601	0.0120	4.5%	13.7%
	**SSP585**	1.480 ± 0.361	0.0837	0.0174	0.0791	0.0146	5.5%	16.1%
GHCN	**SSP126**	0.419 ± 0.189	0.0138	0.0079	0.0130	0.0056	5.8%	29.1%
	**SSP245**	0.734 ± 0.223	0.0358	0.0092	0.0345	0.0070	3.6%	23.9%
	**SSP370**	1.154 ± 0.334	0.0629	0.0139	0.0599	0.0117	4.8%	15.8%
	**SSP585**	1.471 ± 0.352	0.0837	0.0174	0.0788	0.0139	5.9%	20.1%

Supplementary Table 11. Constraint on future yearly increase in total evaporation using constrained future yearly increase in annual precipitation.

	Emission Scenario	Constrained future yearly increase in annual precipitation ± one standard deviation (mm year^{-1})	Future yearly increase in total evaporation before constraint (mm year^{-1})		Future yearly increase in total evaporation after constraint (mm year^{-1})		Overestimated future yearly increase in total evaporation by original CMIP6 outputs (%)	Reduced uncertainty in future yearly increase in total evaporation after constraint (%)
			Mean value	One standard deviation	Mean value	One standard deviation		
HadCRUT4	**SSP126**	0.388 ± 0.178	0.280	0.143	0.266	0.100	5.0%	30.1%
	**SSP245**	0.699 ± 0.222	0.439	0.217	0.400	0.191	8.9%	12.0%
	**SSP370**	1.102 ± 0.332	0.609	0.312	0.530	0.254	13.0%	18.6%
	**SSP585**	1.418 ± 0.350	0.747	0.348	0.658	0.292	11.9%	16.1%
NOAA	**SSP126**	0.388 ± 0.188	0.280	0.143	0.266	0.104	5.0%	27.3%
	**SSP245**	0.699 ± 0.229	0.439	0.217	0.400	0.193	8.9%	11.1%
	**SSP370**	1.102 ± 0.342	0.609	0.312	0.530	0.258	13.0%	17.3%
	**SSP585**	1.418 ± 0.368	0.747	0.348	0.658	0.298	11.9%	14.4%
GISS	**SSP126**	0.424 ± 0.184	0.280	0.143	0.286	0.102	-2.1%	28.7%
	**SSP245**	0.739 ± 0.229	0.439	0.217	0.422	0.197	3.9%	9.2%
	**SSP370**	1.162 ± 0.333	0.609	0.312	0.562	0.256	7.7%	17.9%
	**SSP585**	1.480 ± 0.361	0.747	0.348	0.694	0.290	7.1%	16.7%
GHCN	**SSP126**	0.419 ± 0.189	0.280	0.143	0.283	0.115	-1.1%	19.6%
	**SSP245**	0.734 ± 0.223	0.439	0.217	0.419	0.190	4.6%	12.4%
	**SSP370**	1.154 ± 0.334	0.609	0.312	0.557	0.266	8.5%	14.7%
	**SSP585**	1.471 ± 0.352	0.747	0.348	0.689	0.294	7.8%	15.5%

Supplementary Table 12. Constraint on future yearly decrease in snow cover fraction using constrained future yearly increase in temperature.

	Emission Scenario	Constrained future yearly increase in temperature ± one standard deviation (°C yearly^{-1})	Future yearly decrease in snow cover fraction before emergent constraint (% yearly^{-1})		Future yearly decrease in snow cover fraction after constraint (% yearly^{-1})		Overestimated future yearly decrease in snow cover fraction by original CMIP6 outputs (% year^{-1})	Reduced uncertainty in future yearly decrease in snow cover fraction after constraint (% year^{-1})
			Mean value	One standard deviation	Mean value	One standard deviation		
HadCRUT4	**SSP126**	0.0122 ± 0.0058	−0.0239	0.0151	−0.0143	0.0114	40.2%	24.5%
	**SSP245**	0.0338 ± 0.0075	−0.0452	0.0192	−0.0377	0.0137	16.6%	28.6%
	**SSP370**	0.0584 ± 0.0110	−0.0793	0.0260	−0.0683	0.0190	13.9%	26.9%
	**SSP585**	0.0771 ± 0.0144	−0.1091	0.0344	−0.0918	0.0247	15.9%	28.2%
NOAA	**SSP126**	0.0122 ± 0.0059	−0.0239	0.0151	−0.0143	0.0116	40.2%	23.2%
	**SSP245**	0.0338 ± 0.0075	−0.0452	0.0192	−0.0377	0.0138	16.6%	28.1%
	**SSP370**	0.0584 ± 0.0112	−0.0793	0.0260	−0.0683	0.0193	13.9%	25.8%
	**SSP585**	0.0771 ± 0.0148	−0.1091	0.0344	−0.0918	0.0254	15.9%	26.2%
GISS	**SSP126**	0.0131 ± 0.0057	−0.0239	0.0151	−0.0159	0.0103	33.5%	31.8%
	**SSP245**	0.0346 ± 0.0072	−0.0452	0.0192	−0.0391	0.0127	13.5%	33.9%
	**SSP370**	0.0601 ± 0.0120	−0.0793	0.0260	−0.0710	0.0194	10.5%	25.4%
	**SSP585**	0.0791 ± 0.0146	−0.1091	0.0344	−0.0951	0.0246	12.8%	28.5%
GHCN	**SSP126**	0.0130 ± 0.0056	−0.0239	0.0151	−0.0156	0.0105	34.7%	30.5%
	**SSP245**	0.0345 ± 0.0070	−0.0452	0.0192	−0.0389	0.0127	13.9%	33.9%
	**SSP370**	0.0599 ± 0.0117	−0.0793	0.0260	−0.0706	0.0195	11.0%	25.0%
	**SSP585**	0.0788 ± 0.0139	−0.1091	0.0344	−0.0946	0.0243	13.3%	29.4%

Supplementary Table 13. Probability of extreme snow cover loss in Asia before and after constraining by the constrained future yearly increase in temperature.

Emission Scenario	Future yearly change in snow cover fraction (% $year^{-1}$)	Probability for original CMIP6 outputs	Probability for constrained results
**SSP126**	< −0.045%	8.7%	0.5%
**SSP245**	< −0.076%	5.7%	0.5%
**SSP370**	< −0.119%	6.6%	0.5%
**SSP585**	< −0.159%	7.5%	0.5%

References

1. Pfahl, S., O'Gorman, P. A. & Fischer, E. M. Understanding the regional pattern of projected future changes in extreme precipitation. *Nature. Clim. Change*. **7**, 432-427 (2017).

2. Emori, S. & Brown, S. J. Dynamic and thermodynamic changes in mean and extreme precipitation under changed climate. Geophysical Research Letters, 32(17), L17706 (2005).

3. Ali, H. & Mishra, V. Contributions of dynamic and thermodynamic scaling in subdaily precipitation extremes in India. *Geophys. Res. Lett*. **45**, 2352-2361 (2018).

4. Seeley, J. T. & Romps, D. M. Why does tropical convective available potential energy (cape) increase with warming? *Geophys. Res. Lett*. **42**, 10429-10437 (2015).

5. O'Gorman, P. A. & Schneider, T. (2008). The hydrological cycle over a wide range of climates simulated with an idealized GCM. *J. Climat.* 21(15), 3815-3832.

6. Xu, Z. X., Takeuchi, K. & Ishidaira, H. Correlation between EI Niño–Southern Oscillation (ENSO) and precipitation in south-east Asia and the pacific region. *Hydrol. Process*. **18**, 107-123 (2010).

7. Qu, J., Gong, D., Mao, R., Yang, J. & Li, S. Possible influence of Arctic oscillation on precipitation along the Wast Asian rain belt during boreal spring. *Theor. Appl. Climatol*. **130**, 487-495 (2017).

8. Van der Ent, R. J., Savenije, H. H., Schaefli, B. & Steele-Dunne, S. C. Origin and fate of atmospheric moisture over continents. *Water. Resour. Res.* **46**, W09525 (2010).

9. Chai, Y. et al. Constraining Amazonian land surface temperature sensitivity to precipitation and the probability of forest dieback. *Npj. Clim. Atmos. Sci*. **4**, 6 (2021).

10. Yi, X. S., Li, G. S. & Yin, Y. Y. Spatio-temporal variation of precipitation in the three-river headwater region from 1961 to 2010. *J. Geogr. Sci.* **23**, 447-464 (2013).\

11. Gorodetskaya, I. V., Tremblay, L. B., Liepert, B., Cane, M. A. & Cullather, R. I. The influence of cloud and surface properties on the arctic ocean shortwave radiation budget in coupled models. *J. Climate*. **21**, 866-882 (2008).

12. Wenzel, S., Cox, P. M., Eyring, V. & Friedlingstein, P. (2016). Projected land photosynthesis constrained by changes in the seasonal cycle of atmospheric CO_2. *Nature.* **538**, 499-501.

13. Hall, A., Cox, P., Huntingford, C. & Klein, S. Progressing emergent constraints on future climate change. *Nat. Clim. Chang*. **9**, 269-278 (2019).

14. Chen, J., Brissette, F. P. & Leconte, R. Uncertainty of downscaling method in quantifying the impact of climate change on hydrology. *J. Hydrol*. **401**, 190-202 (2011).

15. Johnson, F. & Sharma, A. Accounting for interannual variability: a comparison of options for water resources climate change impact assessments. *Water. Resour. Res.* **47**, W04508 (2011).

16. Hempel, S., Frieler, K., Warszawski, L., Schewe, J. & Piontek, F. A trend-preserving bias correction–the ISI-MIP approach. *Earth. Syst. Dynam.* **4**, 219-236 (2013).
